# PD-L1 induces autophagy and primary resistance to EGFR–TKIs in EGFR-mutant lung adenocarcinoma via the MAPK signaling pathway

**DOI:** 10.1038/s41419-024-06945-7

**Published:** 2024-08-01

**Authors:** Na Li, Ran Zuo, Yuchao He, Wenchen Gong, Yu Wang, Liwei Chen, Yi Luo, Cuicui Zhang, Zhiyong Liu, Peng Chen, Hua Guo

**Affiliations:** 1 Department of Thoracic Oncology, Lung Cancer Diagnosis and Treatment Center, Tianjin Medical University Cancer Institute and Hospital, Tianjin, 300060 China; 2 National Clinical Research Center for Cancer, State Key Laboratory of Druggability Evaluation and Systematic Translational Medicine, Tianjin’s Clinical Research Center for Cancer, Key Laboratory of Cancer Prevention and Therapy, Tianjin, 300060 China; 3 Department of Oncology, Weihai Municipal Hospital, Cheeloo College of Medicine, Shandong University, Weihai, 264200 China; 4 Department of Integrative Oncology, Tianjin Medical University Cancer Institute and Hospital, Tianjin, 300060 China; 5 Department of Tumor Cell Biology, Tianjin Medical University Cancer Institute and Hospital, Tianjin, 300060 China; 6 Department of Pathology, Tianjin Medical University Cancer Institute and Hospital, Tianjin, 300060 China

**Keywords:** Non-small-cell lung cancer, Prognostic markers

## Abstract

Resistance to epidermal growth factor receptor (EGFR)–tyrosine kinase inhibitors (TKIs) is a significant cause of treatment failure and cancer recurrence in non-small cell lung cancer (NSCLC). Approximately 30% of patients with EGFR-activating mutations exhibit primary resistance to EGFR–TKIs. However, the potential mechanisms of primary resistance to EGFR–TKIs remain poorly understood. Recent studies have shown that increased expression of programmed death ligand-1 (PD-L1) is associated with EGFR–TKIs resistance. Therefore, the present study aimed to investigate the mechanism of PD-L1 in primary resistance to EGFR–TKIs in EGFR-mutant lung adenocarcinoma (LUAD) cells. We found that PD-L1 was associated with poor prognosis in patients with EGFR-mutant LUAD, while the combination of EGFR–TKIs with chemotherapy could improve its therapeutic efficacy. In vitro and in vivo experiments revealed that PD-L1 promoted the proliferation and autophagy and inhibited the apoptosis of LUAD cells. Mechanistic studies demonstrated that upregulation of PD-L1 was critical in inducing autophagy through the mitogen-activated protein kinase (MAPK) signaling pathway, which was beneficial for tumor progression and the development of gefitinib resistance. Furthermore, we found that gefitinib combined with pemetrexed could synergistically enhance antitumor efficacy in PD-L1-overexpression LUAD cells. Overall, our study demonstrated that PD-L1 contributed to primary resistance to EGFR–TKIs in EGFR-mutant LUAD cells, which may be mediated by inducing autophagy via the MAPK signaling pathway. These findings not only help improve the prognosis of patients with EGFR-mutant LUAD but also provide a reference for the research of other cancer types.

## Introduction

Lung cancer is one of the most common malignancies and the leading cause of cancer-related death worldwide [1, 2]. Non-small cell lung cancer (NSCLC) accounts for approximately 85% of all lung cancers [3]. Lung adenocarcinoma (LUAD) is the most common histological subtype of NSCLC, and approximately 15% of Caucasians and 50% of Asians harbor activating epidermal growth factor receptor (EGFR) mutations [4]. Targeting the EGFR signaling pathway has proven to be a successful strategy in NSCLC [5, 6]. However, nearly 30% of patients with EGFR mutations do not benefit from EGFR–tyrosine kinase inhibitors (TKIs) because of primary drug resistance [7]. Therefore, it is imperative to investigate the molecular mechanisms underlying primary drug resistance, develop novel prognostic biomarkers and find effective strategies to overcome such resistance to improve therapeutic benefits. Previous studies have found that the mechanism of primary resistance is complicated and not fully understood [8–11]. Currently, combination therapy is considered a promising strategy not only to overcome resistance and improve therapeutic efficacy but also to reduce the drug doses to attenuate side effects. However, other studies suggested that combination therapy could not bring survival benefits and instead increased adverse drug reactions [12]. How to determine the specific population most likely to benefit from combination therapy under the comprehensive evaluation of clinical efficacy and adverse effects is a major clinical challenge. Therefore, predictive biomarkers are required to guide clinicians’ choice of optimal and effective therapeutic strategies.

Autophagy is a process of intracellular degradation and self-recycling, and it plays a complex dual role in cancer development [13–15]. An increasing number of studies have confirmed that upregulation of autophagy plays a protective role in helping cancer cells avoid drug-induced apoptosis and facilitate tumor progression, invasion and drug resistance [16, 17]. Autophagy is an important factor in acquired resistance to EGFR–TKIs in NSCLC [18, 19]. Sakuma et al. [20] found that upregulation of autophagic flux in EGFR-mutant NSCLC could evade cell apoptosis and confer gefitinib resistance. Therefore, targeting autophagy may be a promising approach to improve the therapeutic efficacy of EGFR–TKIs in advanced NSCLC, which provides a new vision for reducing drug resistance of tumor-targeted therapy.

Programmed death ligand 1 (PD-L1; also known as CD274 or B7-H1) is an immunosuppressive molecule expressed on the surface of tumor cells and immune cell membranes that interacts with programmed death 1 (PD-1) receptors on the surface of T lymphocytes to negatively regulate T cell functions [21]. Recent studies have shown that tumor PD-L1 plays an important role in tumor-intrinsic signaling and survival effects that are unrelated to its immune regulatory functions [22]. PD-L1 can regulate tumor glucose metabolism, autophagy, proliferation, metastasis and drug resistance [23–25]. Accumulating studies have revealed the relationship between the EGFR signaling pathways and PD-L1 expression, although the conclusions remain controversial. Some studies have demonstrated that PD-L1 expression in EGFR-wild-type cells is higher than that in EGFR-mutant NSCLC [26, 27]. Conversely, other studies have found that EGFR mutations upregulate PD-L1 expression in lung cancer [28, 29]. Some studies have reported that the high expression of PD-L1 is associated with EGFR–TKIs resistance [30, 31]. However, the relationship between PD-L1 and primary resistance to EGFR–TKIs and its potential molecular mechanism remain unclear. Recent studies have found that high autophagy levels are generally associated with poor clinical outcomes in certain cancer types. PD-L1 can promote autophagy and chemotherapy resistance of bladder cancer, hepatocellular carcinoma, and gastric cancer [32–34]. Therefore, we hypothesize that PD-L1 may induce primary resistance to EGFR–TKIs in EGFR-mutant LUAD via activation of autophagy.

In this study, we demonstrated that PD-L1 was associated with poor prognosis of patients with EGFR-mutant LUAD receiving EGFR–TKIs, which might serve as a specific prognostic biomarker. In addition, PD-L1 overexpression could promote cell proliferation, inhibit cell apoptosis, and promote autophagy through the MAPK/ERK signaling pathway, leading to primary resistance to EGFR–TKIs. We further confirmed that the combination of gefitinib and chemotherapy could enhance the antitumorigenic effects of gefitinib by inhibiting autophagy in PD-L1-overexpression cells. Our study promises to provide evidence for clinicians to select a reasonable treatment strategy for patients with EGFR-mutant LUAD.

## Materials and methods

### Patients and tissue specimens

A total of 343 treatment naïve patients with inoperable or metastatic stage III and IV LUAD with known EGFR mutation status between March 2016 and April 2022 and with available formalin-fixed paraffin-embedded (FFPE) tumor samples were retrospectively identified at Tianjin Medical University Cancer Institute and Hospital (Tianjin, China). Clinical responses were evaluated using the Response Evaluation Criteria in Solid Tumors guidelines 1.1 (RECIST1.1), responses were classified as: (i) Complete Response (CR): disappearance or reduction to < 10 mm in the short axis for all LN metastases; (ii) Partial Response (PR): > 30% reduction from baseline; (iii) Stable Disease (SD): neither CR, PR, nor PD; and (iv) Progressive Disease (PD): > 20% growth from the minimum sum of diameters during the course and > 5 mm growth [35]. The objective response rate (ORR) was equal to the sum of complete response (CR) plus partial response (PR). The definition of primary resistance was when patients experienced disease progression within 6 months from initial EGFR–TKIs treatment [36]. This study was consistent with the ethical guidelines of the Helsinki Declaration and approved by the Ethics Committee.

### Immunohistochemistry

According to the manufacturer’s instructions, immunohistochemistry (IHC) staining on FFPE specimens of PD-L1 (PD-L1 IHC 22C3 pharmDx Kit) was performed on Dako Automated Link 48 platform (Dako North American, Inc., Carpinteria, USA). The tumor cells were considered positive in the presence of cell membrane staining. PD-L1 expression was quantified using the tumor proportional score (TPS), which was the percentage of viable tumor cells showing partial or complete membrane staining. The cutoffs of PD‐L1 TPS were 1% and 50%; tumors were defined as PD-L1-negative (PD-L1 TPS < 1%), low positive (1% ≤ PD-L1 TPS ≤ 49%), or high positive (PD-L1 TPS ≥ 50%) [37]. All samples in our study were categorized into the following two groups based on PD-L1 expression: positive (TPS ≥ 1%) and negative (TPS < 1%).

### Cell culture and reagents

The EGFR-mutant LUAD cell lines PC9 and HCC827, and EGFR-wild type H1299 cell line were purchased from American Type Culture Collection (ATCC, Manassas, VA, USA). All cells were cultivated in RPMI 1640 medium (Corning, NY, USA), supplemented with 10% fetal bovine serum (FBS; ZETA, South America Origin) and 1% penicillin/streptomycin (PS; HyClone). The incubating temperature was 37 °C, with 5% CO_2_. PD98059 (AbMole Bioscience, M1822, USA) was purchased from AbMole Bioscience. 3-Methyladenine (3-MA) (MCE, HY-19312, USA) was purchased from MedChemExpress. Gefitinib (Selleckchem, S5098, USA) and pemetrexed (Selleckchem, S1135, USA) were purchased from Selleck.

### Cell transfection

To acquire lentiviral particles, packaging plasmids (VSVG and ΔR) and expression plasmids (PD-L1 and vector) were transfected into HEK293T cells using PEI (Polysciences, Warrington, USA). PC9, HCC827 and H1299 cells were infected with lentiviruses to produce stable PD-L1 overexpression (PD-L1) and matched empty vector cell lines under puromycin (Gibco, New York, USA) selection.

### Western blotting and antibodies

Western blotting analysis was conducted as described previously [38]. The following primary antibodies were used: anti-PD-L1 (Rabbit, 1:1000), anti-Bcl-2 (Rabbit, 1:1000), anti-p-JNK (Rabbit, 1:1000), and anti-JNK (Rabbit, 1:1000) from Abmart (Shanghai, China); anti-p-EGFR (Rabbit, 1:1000), anti-EGFR(Rabbit, 1:1000), anti-p-p38 (Rabbit, 1:1000), anti-p38 (Rabbit, 1:1000), anti-p-ERK (Rabbit, 1:1000), anti-ERK (Rabbit, 1:1000), anti-p-AKT (Rabbit, 1:1000), anti-AKT (Rabbit, 1:1000), anti-p-NF-κB (Rabbit, 1:1000), anti-NF-κB (Rabbit, 1:1000), anti-Beclin1 (Rabbit, 1:1000), anti-Atg5 (Rabbit, 1:1000), anti-Atg7 (Rabbit, 1:1000), anti-LC3B (Rabbit, 1:1000), anti-SQSTM1/p62 (Mouse, 1:1000), anti-cleaved PARP (Rabbit, 1:1000), anti-PARP (Rabbit, 1:1000), anti-cleaved caspase 3 (Rabbit, 1:1000), anti-Cyclin D1 (Rabbit, 1:1000), and anti-Cyclin E1 (Rabbit, 1:1000) from Cell Signaling Technology (Danvers, USA); anti-caspase 3 (Rabbit, 1:500) from Proteintech (Chicago, IL, USA); and anti-GAPDH (Mouse, 1:1000) from Bioss (Beijing, China).

### CCK-8 assay

According to the protocol of the Cell Counting Kit-8 (CCK-8) assay, tumor cells were plated in a 96-well plate at 2 × 10^3^ cells per well. Six parallel wells were used for each independent group. And then 10 μL of CCK-8 reagent (Dojindo Laboratories, Kyushu, Japan) was added to each well and incubated with the cells in a 37 °C incubator for 2 h. The optical density (OD) value at 450 nm was evaluated with an enzyme labeling instrument. The cell proliferation curve was drawn by continuous detection on days 0, 1, 2 and 3.

### Colony formation assay

For cell proliferation analysis, 1 × 10^3^ cells were plated in six-well plates and cultured in complete medium. The cells were treated with the indicated agents and incubated at 37 °C in 5% CO_2_ for 14 days. The colonies were fixed with 4% paraformaldehyde (PFA), stained with 0.1% crystal violet, imaged, and counted. In some experiments, cells were treated with 3-MA, PD98059, gefitinib, pemetrexed, or a combination of gefitinib and pemetrexed.

### EDU assay

A BeyoClick^TM^ EdU Cell Proliferation kit (Beyotime, Shanghai, China) was used to detect the cell proliferation ability. Cells were plated in six-well plates with a density of 2 × 10^5^ cells each well. According to the manufacturer’s instructions, cells incubated with 10 μM EDU working solution at 37 °C for 2 h were fixed with 4% PFA for 15 min. EDU solution was added into culture followed by the staining of nuclei with 4′,6-diamidino-2-phenylindole (DAPI; Beyotime, Shanghai). Images were collected under a fluorescence microscope.

### Cell viability assay

Tumor cells were plated in a 96-well plate at a density of 2 × 10^3^ cells per well, grown overnight, and treated with varying drug concentrations for 72 h. The concentrations were as follows: 0, 0.0001, 0.001, 0.01, 0.1, 1, 5, 10, and 20 μM gefitinib, pemetrexed, and combined mixtures. Each well was added with 10 μL of CCK-8 solution, and the OD450 was evaluated with an enzyme labeling instrument after 2 h incubation.

### Drug combination index

The Combination index (CI) calculations was used to assess the drug interaction between gefitinib and pemetrexed derived from the median dose-effect analysis by Chou and Talalay [39]. The CI analysis was calculated by CompuSyn software (ComboSyn, Inc., Paramus, NJ, USA), and the combination effect was indicated as follows: CI < 1 for synergistic effect, CI = 1 for additive effect, and CI > 1 for antagonistic effect.

### Apoptosis and cell cycle assay

For cell apoptosis assay, cells were trypsinized, washed, and stained in the dark with Annexin V-APC and 7-AAD (BD Pharmingen, New Jersey, USA) for 30 min. Finally, the cells were evaluated by BD FCSCanto II (Becton, Dickinson and Company, New Jersey, USA). The percentage of apoptotic cells was assessed by flow cytometry. For cell cycle assay, PC9 and HCC827 cells were fixed by 95% ice-cold ethanol and incubated at 4 °C overnight. Following centrifugation and washing, the cells were stained with propidium iodide (PI; BD Pharmingen, New Jersey, USA) in the dark for 30 min. The cell samples were assessed on a FACS Aria flow cytometer (BD) with CellQuest software. The percentage of apoptotic cells and the cell cycle results were evaluated with FlowJo software.

### Cell scratch assay

When the cells seeded in six-well plates (2 × 10^6^ cells/well) reached confluence, an equal-width scratch was made across the plate by using a 10 μL pipette tip. The detached cells were removed with PBS and incubated with FBS-free culture medium alone or containing different reagents of gefitinib, pemetrexed, or combined mixtures. Images of the scratched cells were obtained at a suitable time (0, 3, 6, 9, 12, 24, or 48 h) with a microscope at 4 × magnification. Data were shown as the mean ± SD.

### Migration and invasion assays

Cell migration and invasion were detected using Transwell chambers (Corning). A total of 1 × 10^5^ cells in FBS-free medium was added to the upper chambers, and 600 μL of medium containing 20% FBS was loaded to the lower chamber. For the invasion assay, Matrigel-coated Transwell chambers were incubated for more than 2 h at 37 °C. The plates were incubated at 37 °C and 5% CO_2_ for 24 h. In some experiments, cells were pre-treated with gefitinib, pemetrexed, or combined mixtures, followed by an incubation period of 72 h. Subsequently, the cells were digested and resuspended in FBS-free medium. And 200 μL of cell suspension (1 × 10^5^ cells) was added to the upper chambers of a Transwell system. In both assays, the cells on the bottom were fixed with 4% PFA for 20 min, stained with 0.1% crystal violet and observed under a microscope.

### Immunofluorescence

Cells were seeded in six-well plates, fixed in 4% PFA, blocked with 5% bovine serum albumin (BSA), and incubated with LC3B primary antibody, followed by incubation with fluorescently labeled secondary antibodies. The nuclei were stained with DAPI, and cells were visualized using fluorescence microscopy.

### Transmission electron microscopy (TEM)

TEM analysis was performed as described in previous studies [40]. In brief, cells were fixed with 2.5% glutaraldehyde at 4 °C for 4 h, embedded, and sliced (LEICA EM UC7). Cell morphology was observed under TEM (HITACHI, Tokyo, Japan), and the images were captured by Wuhan Servicebio Technology CO., LTD and analyzed using image processing software (ImageJ, National Institutes of Health, Bethesda, MD, USA).

### Gene ontology (GO) enrichment analysis

Gene expression data for patients with lung cancer were downloaded from The Cancer Genome Atlas (TCGA, https://portal.gdc.cancer.gov/) database. We used the median value of PD-L1 expression to divide patients with LUAD into low- and high-expression groups. Differential gene analyses based on the groups were performed using the edgeR package. Differentially expressed genes (DEGs) were screened out based on the criteria of foldchange > 2 and *p*-value < 0.05. A total of 1148 DEGs were identified, of which 781 were upregulated and 367 were downregulated in the low CD274 expressed group compared with the high CD274 expressed group. The enrichGO function of R package clusterProfiler was utilized to perform Gene ontology (GO) enrichment analysis on these 1148 DEGs with pvalueCutoff = 0.05 and qvalueCutoff = 0.2. Significant enriched pathways were visualized using the ggplot2 package.

### Autophagic flux detection

The cells were transfected with lentivirus expressing the StubRFP-sensGFP-LC3 fusion protein (Genechem, Shanghai, China) following the manufacturer’s protocol. After transfection with mRFP-GFP-LC3, autophagosomes were labeled yellow (mRFP and GFP), whereas autolysosomes were labeled red (mRFP only). Finally, cells were photographed by using a confocal microscope.

### Animal models

Female BALB/c nude mice (4–5 weeks old) were purchased from SPF Biotechnology (Beijing, China) for xenograft animal assays. For tumor growth assays, the mice were randomly divided into three groups (PC9 vector, PC9 PD-L1, and PC9 PD-L1 + PD98059; *n* = 6 per group). The prepared PC9 vector/ PD-L1 tumor cells (5 × 10^6^) in 100 μL of PBS were injected subcutaneously. When the tumor volumes reached approximately 80–100 mm^3^, PD98059 or solvent (10 mg/kg, intraperitoneally, daily) were given. For drug sensitivity experiments, PC9 PD-L1 tumor cells (5 × 10^6^) in 100 μL of PBS were injected subcutaneously into each mouse. When the tumor volumes reached 100–150 mm^3^, the mice were randomly allocated into four groups (*n* = 6 per group): control group (solvent alone), gefitinib group (50 mg/kg gefitinib, oral gavage, five times a week), pemetrexed group (100 mg/kg pemetrexed, intraperitoneally, three times a week), and combination group (gefitinib and pemetrexed were administered according to the aforementioned regimens). Tumor volume was measured every 2 days, and animal weight was measured every 4 days. Tumor growth was analyzed by measuring tumor length (a) and width (b), and it was calculated using the following formula: volume (mm^3^) = ab^2^/2. Mice were humanely sacrificed on days 18–20, and tumor weight was recorded. The harvested tumor tissues were fixed in 4% paraformaldehyde, stained with hematoxylin and eosin (H&E). Finally, the IHC was performed to detect the protein level of PD-L1 under different treatments according to previously described methods.

### Statistical analysis

The Kaplan–Meier method and multivariate Cox method were used to evaluate the survival curves and independent risk factors. The categorical variables were compared by Chi-square test or Fisher’s exact test. Multiple logistic regression was performed to investigate the risk factors associated with short-term effectiveness. All numerical data were presented as the mean ± standard error (SD). Statistical analysis was performed by a two-tailed Student’s t-test or one-way ANOVA. Statistical significance was defined as *P* < 0.05. All results were repeated at least three times independently. All statistical analyses were performed using GraphPad Prism 8 (GraphPad, San Diego, CA, USA) and SPSS 26.0 software (SPSS, Chicago, IL, USA).

## Results

### PD-L1 is associated with poor prognosis in patients with EGFR-mutant LUAD

**Fig. 1:**
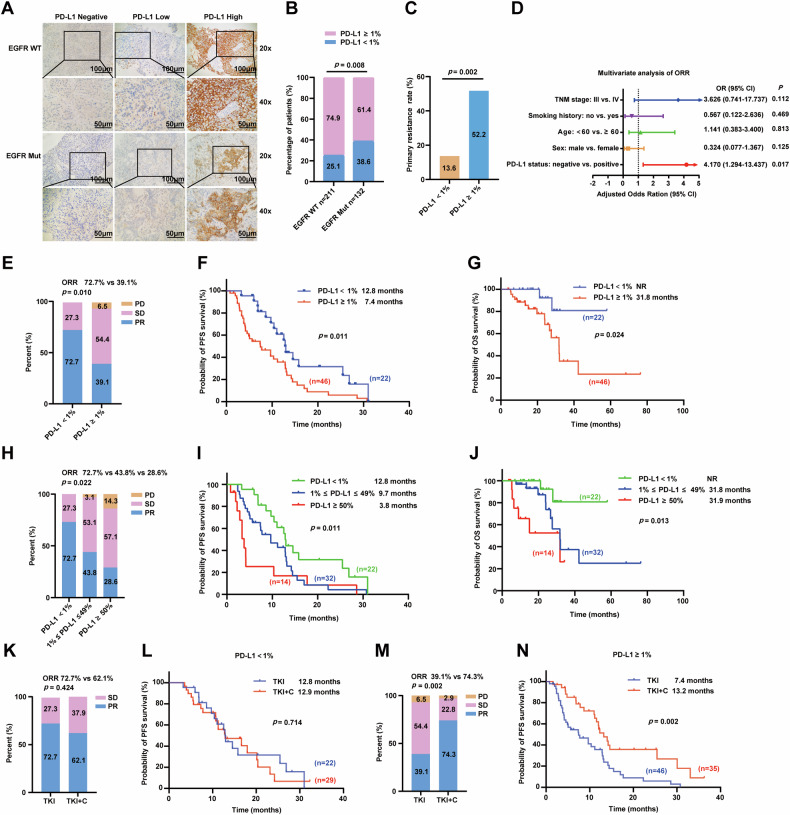
PD-L1 expression is decreased in EGFR-mutant LUAD tissue samples and predicts poor survival in EGFR-mutant LUAD patients. **A** Characteristic IHC images of PD-L1 in EGFR-wild-type and EGFR-mutant LUAD tissues. (PD-L1 negative: TPS < 1%; PD-L1 low: 1% ≤ TPS ≤ 49%; PD-L1 high: TPS ≥ 50%) (scale bar, 100 μm; magnification, 20 × and 40 ×). **B** The percentages of patients with positive expression and negative expression of PD-L1 were assigned according to EGFR mutation status. **C** The primary resistance (PFS < 6 months) in PD-L1-negative group and PD-L1-positive group treated with EGFR–TKIs monotherapy. **D** Risk factors affecting ORR of EGFR–TKIs were analyzed by multivariate logistic regression analysis. **E** The ORR of EGFR–TKIs in PD-L1-negative group and PD-L1-positive group. **F**, **G** The Kaplan-Meier survival curve validating the correlation between PD-L1 expression and EGFR-mutant LUAD patients’ PFS and OS treated with EGFR–TKIs monotherapy. **H** The ORR of EGFR–TKIs in PD-L1-negative group, PD-L1-low group and PD-L1-high group. **I**, **J** The Kaplan-Meier survival curve validating the correlation between PD-L1 expression levels and EGFR-mutant LUAD patients’ PFS and OS treated with EGFR–TKIs monotherapy. **K** The ORR of EGFR-TKIs with or without chemotherapy in PD-L1-negative patients. **L** The Kaplan-Meier survival curve of PFS for PD-L1-negative patients treated with EGFR–TKIs with or without chemotherapy. **M** The ORR of EGFR–TKIs with or without chemotherapy in PD-L1-positive patients. **N** The Kaplan-Meier survival curve of PFS for PD-L1-positive patients treated with EGFR–TKIs with or without chemotherapy. EGFR WT, EGFR-wild-type; EGFR Mut EGFR-mutant, OR Odds Ration, ORR Objective response rate, PR partial response, SD stable disease, PD progressive disease, PFS progress-free survival, OS overall survival, TKI Tyrosine kinase inhibitor, C chemotherapy, NR Not reached. Data were presented as mean ± SD.

To investigate whether a relationship exists between PD-L1 expression and prognosis of patients with EGFR-mutant LUAD, a cohort of 343 LUAD patients was collected. PD-L1 was scored using TPS with cutoffs of less than 1% as negative, between 1% and 49% as low expression, and 50% and higher as high expression (Fig. 1A). Patients with EGFR wild-type LUAD showed a higher PD-L1 expression than those with EGFR mutations (Fig. 1B). Among 68 EGFR-mutant patients treated with EGFR–TKIs monotherapy, the incidence of primary resistance was higher in the PD-L1-positive group than in the PD-L1-negative group (Fig. 1C), and multivariate analyses showed that PD-L1 was an independent predictor for poor ORR (Fig. 1D and Supplementary Table 1). As shown in Fig. 1E, the ORR was significantly higher in the PD-L1-negative group than in the PD-L1-positive group. Furthermore, Kaplan–Meier analysis showed that positive PD-L1 expression significantly shortened progression-free survival (PFS) (Fig. 1F) and overall survival (OS) (Fig. 1G) compared with negative PD-L1 expression. Multivariate COX regression analysis revealed that PD-L1 expression was an independent risk factor for PFS in patients treated with EGFR–TKIs monotherapy (Table 1). In addition, in the 68 EGFR–TKIs monotherapy population, high PD-L1 expression was associated with a significantly decreased ORR compared with low or negative expression (Fig. 1H). High PD-L1 expression was also associated with a significantly shorter PFS (Fig. 1I) and OS (Fig. 1J) compared with low or negative PD-L1 expression. Furthermore, of the 132 EGFR-mutant patients, 51 patients were enrolled in the PD-L1-negative group and 81 patients were enrolled in the PD-L1-positive group. The combination of EGFR–TKIs and chemotherapy did not improve the ORR (Fig. 1K) and prolong the PFS of PD-L1-negative patients compared with TKIs monotherapy (Fig. 1L), but it could significantly improve the ORR (Fig. 1M) and prolong the PFS of PD-L1-positive patients (Fig. 1N). These findings indicated that PD-L1 might be a key factor in EGFR–TKIs resistance and served as a promising prognostic biomarker for EGFR-mutant LUAD patients. Collectively, these results suggested that PD-L1 might play a critical role in the progression of EGFR-mutant LUAD.

**Table 1:** Univariate and multivariate analysis for PFS in EGFR–TKIs monotherapy cohort.

Characteristics	Univariate analysis HR (95% CI)	*p*-value	Multivariate analysis HR (95% CI)	*p*-value
Sex male/female	1.398 (0.817–2.392)	0.222		
Age ≥ 60/ < 60 years old	1.008 (0.584–1.740)	0.976		
Smoking history yes/no	1.394 (0.798–2.435)	0.244		
TNM stage IV/III	1.514 (0.735–3.119)	0.260		
ECOG ≥ 2/0-1	14.508 (3.445–61.096)	< 0.001	17.101 (3.814–76.678)	< 0.001
Brain metastasis yes/no	0.603 (0.256–1.423)	0.248		
Liver metastasis yes/no	1.036 (0.464–2.310)	0.932		
Bone metastasis yes/no	1.043 (0.598–1.819)	0.883		
PD-L1 positive/negative	2.147 (1.178–3.915)	0.013	2.222 (1.216–4.060)	0.009

*HR* Hazard ratio, *TNM* Tumor node metastasis, *ECOG* Eastern Cooperative Oncology Group.

### PD-L1 promotes proliferation and motility of EGFR-mutant LUAD cells and inhibits cell apoptosis

**Fig. 2:**
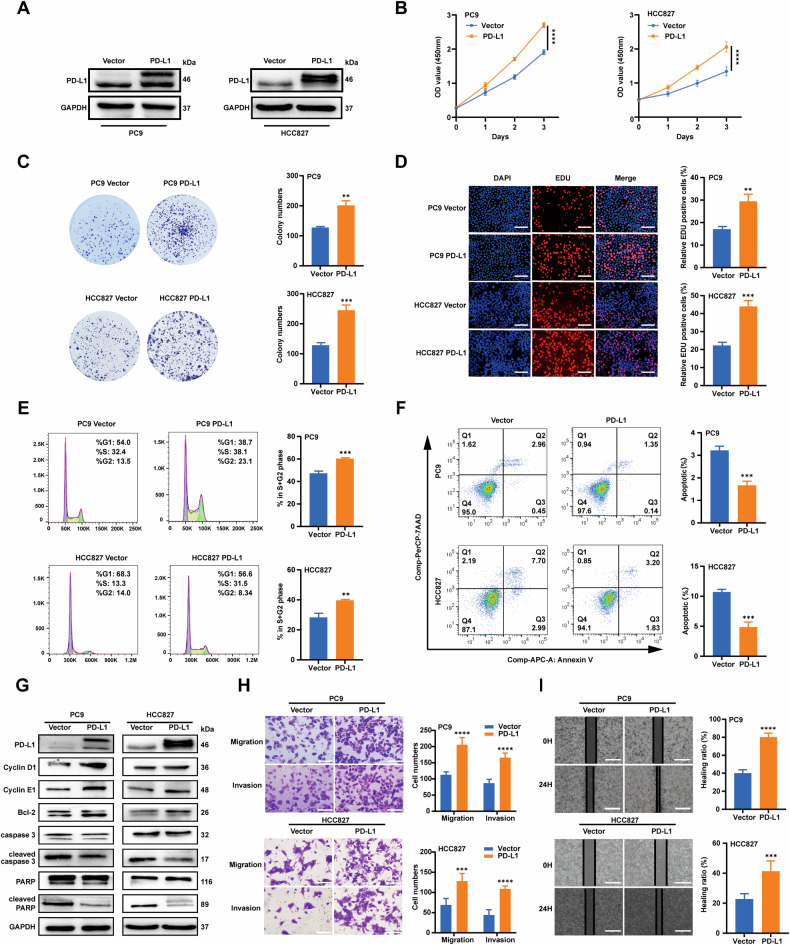
PD-L1 promotes proliferation and motility of EGFR-mutant LUAD cells and inhibits cell apoptosis. **A** Western blotting verification of PD-L1 overexpression (PD-L1) in PC9 and HCC827 cells. **B** CCK-8 assay revealing the impacts of PD-L1 on PC9 and HCC827 cell growth. **C**. Colony formation assay showing the effects of PD-L1 on PC9 and HCC827 cell growth. **D** EDU assay revealing the effects of PD-L1 on PC9 and HCC827 cell proliferative abilities (scale bar, 150 μm). **E** Cell cycle assay revealing the differences of the proliferation index (sum of S and G2/M phases proportion) between the PD-L1 and vector groups of PC9 and HCC827 cell lines. **F** Cell apoptosis assay revealing the proportions of apoptotic PC9 and HCC827 cells in the PD-L1 and vector groups using Annexin V/7-AAD double staining. **G** The protein levels of PD-L1, cell cycle-related and apoptosis-related markers in PC9 and HCC827 cells were assessed by Western blotting. **H** Cell migration and invasion in the PD-L1 and vector groups of PC9 and HCC827 cells were measured by Transwell assay (scale bar, 150 μm; magnification, 20 ×). **I**. Wound-­healing assay comparing the cell migration differences among the PD-L1 and vector groups of PC9 and HCC827 cells (scale bar, 750 μm). The analyses were repeated three times. Data were presented as mean ± SD. *p < 0.05, **p < 0.01, ***p < 0.001, ****p < 0.0001.

Stable PD-L1 overexpression (PD-L1) and vector cell lines were established in PC9 and HCC827 cells (Fig. 2A). The results showed that PD-L1 overexpression increased cell proliferation (Fig. 2B–D). Furthermore, cell cycle analysis showed that PD-L1 overexpression led to a decrease in the proportion of PC9 cells in the G1 phase and an increase in the sum of cells in S and G2/M phases, resulting in a higher proliferation index. Similar results were observed in HCC827 cells, suggesting that PD-L1 might promote cell proliferation (Fig. 2E). In addition, Cyclin D1 and Cyclin E1, critical regulators of G1 to S phase, were upregulated (Fig. 2G). These results indicated that PD-L1 promoted cell cycle progression by upregulating Cyclin D1 and Cyclin E1 levels, thereby accelerating cell proliferation. Flow cytometry showed that PD-L1 overexpression reduced cell apoptosis (Fig. 2F). Furthermore, the expression of apoptosis-related proteins, such as Bcl-2, cleaved PARP, and cleaved caspase 3 was assessed by Western blotting. Overexpression of PD-L1 led to increased Bcl-2 expression but resulted in decreased cleaved PARP and cleaved caspase 3 expression (Fig. 2G). In addition, the chemotaxis and Transwell assays showed that PD-L1 upregulation enhanced the migration and invasion capacities of EGFR-mutant LUAD cells (Fig. 2H, I). Collectively, these results indicated that PD-L1 could promote the malignant biological behaviors of EGFR-mutant LUAD cells.

### PD-L1 inhibits apoptosis by promoting autophagy in EGFR-mutant LUAD cells

**Fig. 3:**
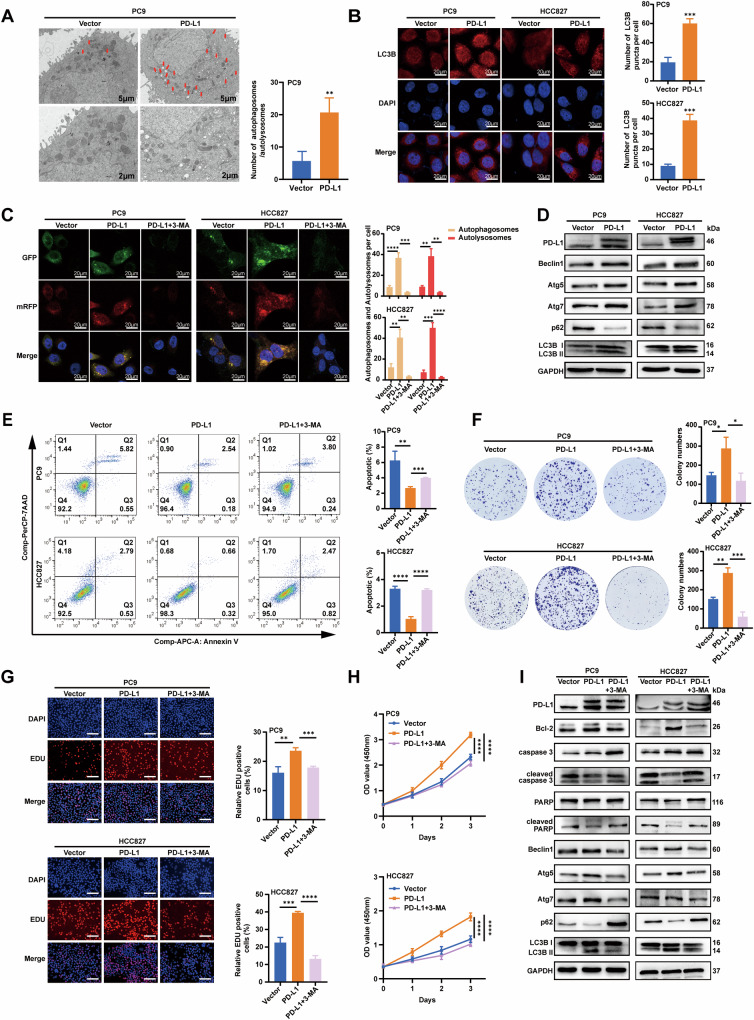
PD-L1 inhibits apoptosis by promoting autophagy in EGFR-mutant LUAD cells. **A** Autophagy was evaluated using TEM in PC9 PD-L1 and vector cells (left). Autophagosomes and autolysosomes were indicated by arrows (scale bar, 5 μm (upper) and 2 μm (lower)). The number of autophagosomes/autolysosomes was quantified (right). **B** Immunofluorescence assay revealing the effects of PD-L1 on PC9 and HCC827 cell autophagy (scale bar, 20 μm). **C** PC9 and HCC827 cells transfected with mRFP-GFP-LC3 lentivirus. PC9 PD-L1 and HCC827 PD-L1 cells were pretreated with 3-MA. Fluorescence microscopy was used to acquire images and the average number of yellow dots (autophagosomes) or red-only dots (autolysosomes) in the merged images per cell was quantified (scale bar, 20 μm). **D** Western blotting analysis of autophagy-related protein levels in PD-L1 and vector groups of PC9 and HCC827 cells. **E**. Apoptotic rate was measured using Annexin V/7-AAD staining in cells as in (**C**). **F**–**H** The viability of cells as in (**C**) was assessed by colony formation assay (**F**), EDU assay (**G**) and CCK-8 assay (**H**) (scale bar, 150 μm). **I** The apoptosis and autophagy-related protein levels were assessed by Western blotting in cells as in (**C**). The analyses were repeated three times. Data were presented as mean ± SD. *p < 0.05, **p < 0.01, ***p < 0.001, ****p < 0.0001.

Autophagy has been shown to promote tumor growth and regulate migration and invasion in several pathways [41]. To further explore the molecular mechanism of PD-L1 regulating the malignant progression of lung cancer, we performed a GO enrichment analysis on the differentially expressed genes (DEGs) in two groups of patients with different PD-L1 levels obtained from the TCGA database. The results showed that PD-L1 was closely related to autophagy (Supplementary Fig. 1). Transmission electron microscopy (TEM) data showed that the number of autophagosomes and autolysosomes significantly increased in PC9 PD-L1 cells (Fig. 3A). Immunofluorescence assay also supported our conclusion, as the RFP-LC3B puncta (autophagosomes) were more abundant in PD-L1-overexpression PC9 and HCC827 cells (Fig. 3B). To further investigate the effect of PD-L1 on autophagy, we used the mRFP-GFP-LC3B double-fluorescence system. The results showed that the number of yellow dots (autophagosomes) and red-only dots (autolysosomes) increased in PD-L1-overexpression cells, but this effect was reversed by autophagy inhibitor 3-MA (Fig. 3C). We also detected the expression of autophagy-related proteins by Western blotting. Overexpression of PD-L1 resulted in increased levels of LC3B II, Beclin1, Atg5, and Atg7 and decreased the level of p62 (Fig. 3D). Taken together, these results indicated that PD-L1 activated autophagy and promoted autophagic flux.

Previous studies have shown that autophagy and apoptosis interact in a complex manner to promote or inhibit each other [42]. To investigate the crosstalk between PD-L1-induced autophagy and apoptosis, we used 3-MA to inhibit autophagy in PD-L1-overexpression cells and found that the apoptosis rate increased and cell viability was suppressed (Fig. 3E–H). Furthermore, Western blotting showed the same results as depicted in Fig. 3I. In conclusion, these data showed that PD-L1 inhibited apoptosis by promoting autophagy.

### PD-L1 inhibits cell apoptosis by promoting autophagy via the MAPK signaling pathway in EGFR-mutant LUAD cells

**Fig. 4:**
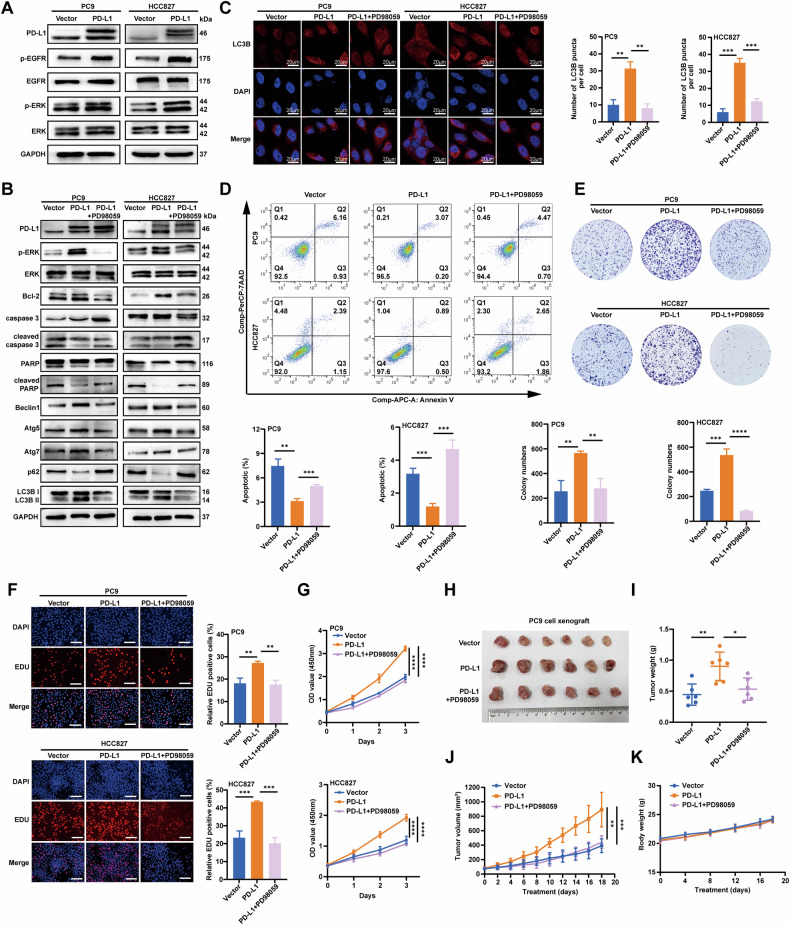
PD-L1 inhibits cell apoptosis by promoting autophagy via the MAPK signaling pathway in EGFR-mutant LUAD cells. **A** The effects of PD-L1 overexpression on EGFR downstream signaling pathways activation were verified by Western blotting in PC9 and HCC827 cells. **B** The protein levels of p-ERK, ERK, apoptosis and autophagy-related proteins were assessed by Western blotting. PC9 PD-L1 and HCC827 PD-L1 cells were pretreated with MER/ERK pathway inhibitor PD98059. **C** PC9 and HCC827 cells as in (**B**) were immunostained with antibodies against LC3B (scale bar, 20 μm). **D** Apoptotic rate was measured using Annexin V/7-AAD staining in cells as in (**B**). **E**, **G** The viability of cells as in (**B**) was assessed by colony formation assay (**E**), EDU assay (**F**) and CCK-8 assay (**G**) (scale bar, 150 μm). **H**–**K** Images of tumors from nude mice (**H**), tumor weights (**I**), the tumor growth rates (**J**) and mice body weights (**K**) in vector, PD-L1 and PD-L1 + PD98059 groups. The analyses were repeated three times. Data were presented as mean ± SD. *p < 0.05, **p < 0.01, ***p < 0.001, ****p < 0.0001.

Aberrant activation of the EGFR downstream signaling pathways, including PI3K/AKT and MAPK/ERK, is the key driver of LUAD. PD-L1 has been reported to be associated with the EGFR signaling pathway. To explore the molecular mechanism of PD-L1 in the induction of autophagy, we investigated the relationship between PD-L1 and the EGFR signaling pathway. Phosphorylation of ERK (p-ERK) significantly increased in PD-L1-overexpression PC9 and HCC827 cells compared with vector cells (Fig. 4A). However, no significant changes were observed in p-p38, p-JNK, p-AKT, and p-NF-κB protein levels (Supplementary Fig. 2). These results demonstrated that PD-L1 functions through the MAPK/ERK pathway, rather than the PI3K/AKT signaling pathway. To investigate whether the MAPK signaling pathway is involved in PD-L1-mediated apoptosis and autophagy, we treated PD-L1-overexpression PC9 and HCC827 cells with the MEK/ERK pathway inhibitor PD98059. Western blotting demonstrated that PD98059 effectively inhibited PD-L1-induced activation of p-ERK; significantly decreased the levels of Beclin1, Atg5, Atg7, and LC3B II; and increased the level of p62. Furthermore, PD98059 decreased the level of Bcl-2 and increased the levels of cleaved caspase 3 and cleaved PARP in PD-L1-overexpression cells (Fig. 4B). Notably, when the MAPK signaling pathway was inhibited by PD98059, the effects of PD-L1-induced autophagy, inhibited apoptosis and promoted cell proliferation of LUAD cells were also reversed (Fig. 4C–G). We observed the same results in the EGFR-wild type H1299 cell line (Supplementary Fig. 3A–G). All these results indicated that PD-L1 regulated autophagy and apoptosis of LUAD cells through the EGFR downstream MAPK signaling pathway.

To further verify the oncogenic activity of PD-L1 in vivo, we implanted PC9 PD-L1 and vector cells subcutaneously into BALB/c mice (5 × 10^6^ cells/mouse). We then investigated whether targeting the MAPK/ERK pathway can inhibit the rapid tumor growth induced by PD-L1 in vivo. Specifically, PD98059 (10 mg/kg) or solvent was injected intraperitoneally daily for 18 days. The tumor weight and volume were significantly higher in the PD-L1 group than in the vector group. Notably, PD98059 could significantly reduce the tumor volume and weight of xenograft tumors in the PD-L1 group (Fig. 4H–J), without serious toxicity as there was no significant changes in body weight (Fig. 4K). These results indicated that PD-L1 significantly promoted the proliferation of EGFR-mutant LUAD in vivo, which may be dependent on the activation of the MAPK/ERK pathway, and the pathway inhibitors could inhibit tumor growth in vivo.

### PD-L1 contributes to EGFR–TKIs resistance, and chemotherapy is synergistic with EGFR–TKIs in EGFR-mutant LUAD cells

**Fig. 5:**
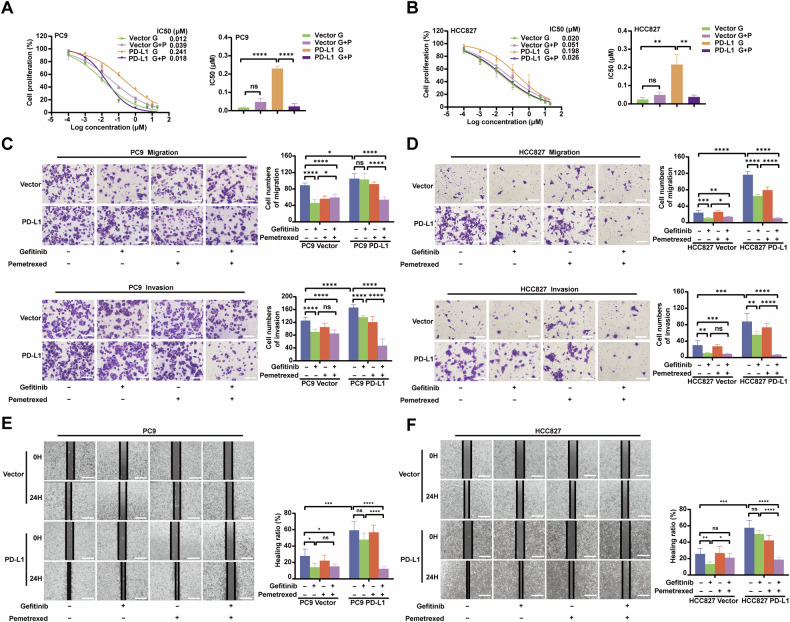
PD-L1 is involved in EGFR–TKIs resistance, chemotherapy is synergistic with EGFR–TKIs to inhibit the migration and invasion in EGFR-mutant LUAD cells. **A**, **B** CCK-8 analysis of cell viability was used to calculate the IC50 values of gefitinib (G) with or without pemetrexed (P) in PC9 (**A**) and HCC827 (**B**) cells under drug treatments for 72 h. **C** Cell migration and invasion of PC9 PD-L1 and vector cells were measured by Transwell assay. The different cells were pretreated with gefitinib, pemetrexed or combined treatment for 72 h (scale bar, 150 μm; magnification, 20 ×). **D** Cell migration and invasion of HCC827 PD-L1 and vector cells were measured by Transwell assay. The different cells were pretreated with gefitinib, pemetrexed or combined treatment for 72 h (scale bar, 150 μm; magnification, 20 ×). **E** Wound-healing assay comparing the cell migration differences among PC9 PD-L1 and vector cells under gefitinib, pemetrexed or combined treatment for 24 h (scale bar, 750 μm). **F** Wound-­healing assay comparing the cell migration differences among HCC827 PD-L1 and vector cells under gefitinib, pemetrexed or combined treatment for 24 h (scale bar, 750 μm). The analyses were repeated three times. Data were presented as mean ± SD. ns: *p* > 0.05, *p < 0.05, **p < 0.01, ***p < 0.001, ****p < 0.0001.

**Fig. 6:**
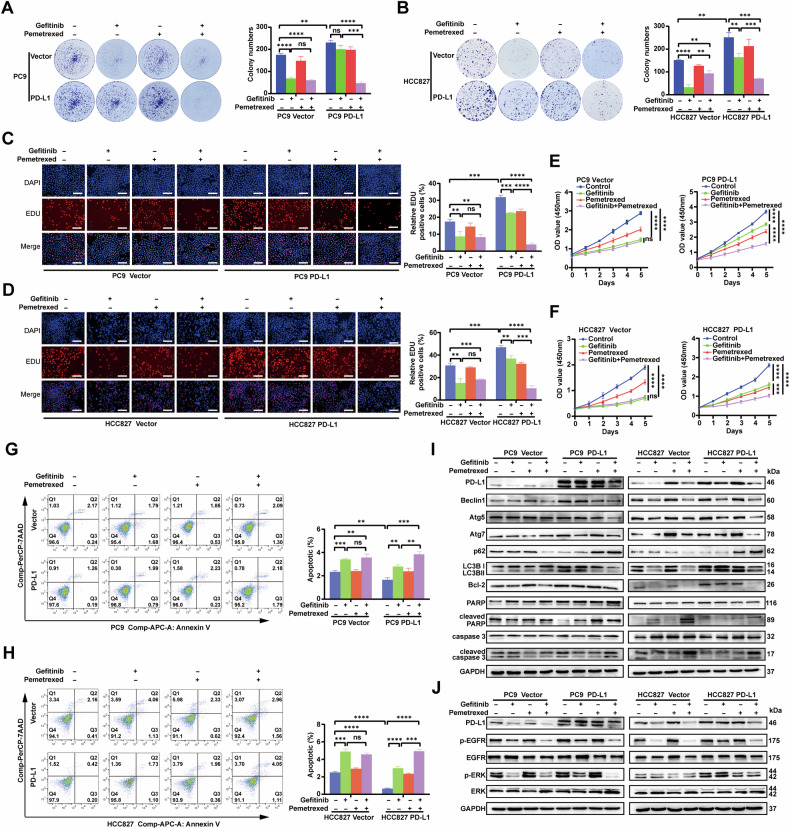
Chemotherapy synergizes with EGFR–TKIs for antitumor efficacy in PD-L1 overexpression EGFR-mutant LUAD cells. **A**, **B** Colony formation assay of PC9 vector, PC9 PD-L1, HCC827 vector and HCC827 PD-L1 cells under gefitinib, pemetrexed or combined treatment for 14 days. **C**, **D** The viability of cells as in (**A**, **B**) pretreated with gefitinib, pemetrexed or combined treatment for 72 h was analyzed by EDU assay (scale bar, 150 μm). **E**, **F** The viability of cells as in (**A**, **B**) treated with gefitinib, pemetrexed or combined treatment was analyzed by CCK-8 assay for 5 days. **G**, **H** Annexin V/7-AAD flow cytometry analysis of cells under gefitinib, pemetrexed or combined treatment for 72 h. **I**, **J** The protein levels of apoptosis-related markers, autophagy-related markers, p-EGFR, EGFR, p-ERK and ERK in different cells under gefitinib, pemetrexed or combined treatment for 72 h. The analyses were repeated three times. Data were presented as mean ± SD. ns: *p* > 0.05, *p < 0.05, **p < 0.01, ***p < 0.001, ****p < 0.0001.

Preclinical and clinical studies have shown that high expression of PD-L1 is associated with acquired EGFR**–**TKIs resistance. Preclinical studies have shown that the combination of pemetrexed and EGFR**–**TKIs has synergistic effects [43]. The NEJ009 trial also showed that EGFR**–**TKIs in combination with chemotherapy significantly improved PFS [44]. To determine whether PD-L1 mediates primary resistance to EGFR**–**TKIs and whether gefitinib combined with pemetrexed has a synergistic anticancer effect, PD-L1-overexpression PC9 and HCC827 cells were exposed to different concentrations of gefitinib with or without pemetrexed for 72 h, and cell viability was measured by CCK-8 assay. As shown in Fig. 5A, B, the half-maximal inhibitory concentration (IC50) for gefitinib dramatically increased in PD-L1-overexpression cells, indicating that PD-L1 overexpression significantly reduced the sensitivity to gefitinib in EGFR-mutant LUAD, whereas the combination treatment rescued PD-L1-induced gefitinib resistance. The CI values showed significant synergism between gefitinib and pemetrexed in PD-L1 overexpression cells, but no apparent synergistic effect was found in the vector group (Supplementary Fig. 4A, B). Furthermore, compared with gefitinib group, gefitinib combined with pemetrexed produced a stronger inhibition of migration and invasion of PD-L1-overexpression cells (Fig. 5C–F). The same trend was also seen in EGFR-wild type H1299 cell line (Supplementary Fig. 3H–J). Notable, PD-L1 overexpression cells attenuated the inhibitory effects of gefitinib on cell proliferation and promotion of cell apoptosis as compared with the vector cells, but the combined treatment could significantly inhibit cell proliferation and promote apoptosis (Fig. 6A–H). Western blotting further showed that gefitinib did not significantly affect the expression of PD-L1, cleaved caspase 3, cleaved PARP, and Bcl-2 in PD-L1-overexpression cells. Gefitinib in combination with pemetrexed significantly decreased the expression of PD-L1 and Bcl-2, but increased the expression of cleaved caspase 3 and cleaved PARP. Similarly, combination treatment was more effective than gefitinib in reducing the expression of Beclin1, Atg5, Atg7, and LC3B II in PD-L1 cells (Fig. 6I). The levels of p-EGFR and p-ERK were dramatically downregulated after combination therapy, whereas no significant change was observed with gefitinib alone in PD-L1-overexpression cells (Fig. 6J). Our results suggested that PD-L1 might play a role in promoting autophagy and inhibiting apoptosis, which was responsible for the generation of primary resistance to EGFR–TKIs, and the combination therapy exerted a synergistic antitumor effect by inhibiting the activation of the MAPK/ERK signaling pathway.

### Chemotherapy enhances the anti-tumor effect of gefitinib in PD-L1-overexpression LUAD in vivo

**Fig. 7:**
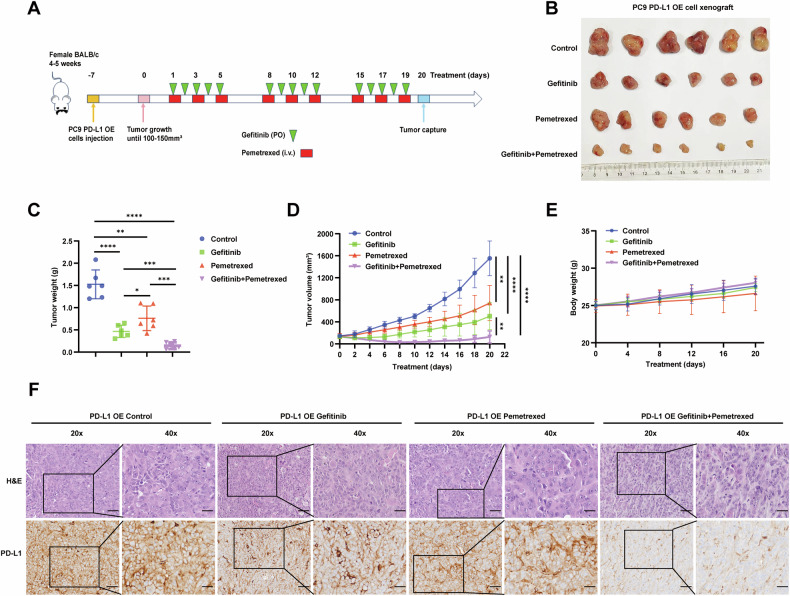
Chemotherapy and gefitinib synergistically suppress tumor growth in PD-L1-overexpression EGFR-mutant LUAD in vivo model. **A** PC9 PD-L1-overexpression (OE) cells were injected subcutaneously into BALB/c mice. The tumor-bearing mice were randomly divided into four groups: Control group (solvent alone), Gefitinib group (50 mg/kg gefitinib, oral gavage, five times a week), Pemetrexed group (100 mg/kg pemetrexed, intraperitoneally, three times a week), and Combination group (both gefitinib and pemetrexed). **B** Representative images of tumors after inoculation using PC9 PD-L1 OE cells treated with gefitinib, pemetrexed, or the combination. **C**, **D** The tumor weights and volumes in the four groups after 20 days treatment. **E** Body weights were recorded every 4 days after treatment. **F** IHC analysis of PD-L1 protein expression was performed in the xenograft model treated as indicated above. (scale bar, 100 μm; magnification, 20 × and 40 ×). Data were presented as mean ± SD. *p < 0.05, **p < 0.01, ***p < 0.001, ****p < 0.0001.

To further verify the synergistic effect of combined therapy in vivo, we subcutaneously inoculated PC9 cells with stable PD-L1-overexpression into BALB/c mice. When the tumor size reached an average of 100–150 mm^3^, the mice were randomly divided into four groups: control, gefitinib, pemetrexed and gefitinib +pemetrexed. As shown in Fig. 7A–E, gefitinib and pemetrexed effectively inhibited xenograft tumor growth compared with the control group. Notably, the combination treatment further reduced tumor volume compared with the other three treatment groups, without serious toxicity in terms of body weight change. In addition, IHC staining for PD-L1 revealed that gefitinib combined with pemetrexed significantly downregulated the expression of PD-L1 in xenograft tumor tissues (Fig. 7F), indicating that the combination treatment presumably reversed gefitinib-resistance in PD-L1 overexpression cells via inhibiting the expression of PD-L1. These results indicated that overexpression of PD-L1 decreased the antitumor sensitivity of EGFR–TKIs in vivo, whereas EGFR–TKIs and chemotherapy had a significant synergistic effect, thus improving the efficacy.

**Fig. 8:**
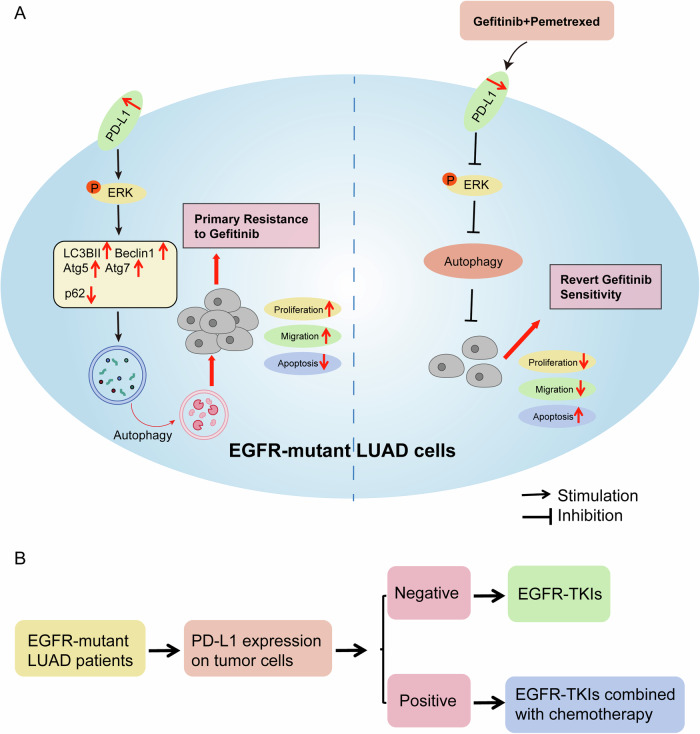
Proposed mechanistic scheme shows the role of PD-L1 in inducing autophagy and gefitinib resistance in EGFR-mutant LUAD. **A** PD-L1 induces autophagy and gefitinib resistance, and pemetrexed synergizes with gefitinib to inhibit PD-L1 expression and MAPK/ERK signaling to decrease autophagy, which enhances the antitumor efficacy in EGFR-mutant LUAD cells. **B** The novel treatment strategy is based on the expression of PD-L1 in EGFR-mutant LUAD patients.

Based on our findings, the potential mechanism whereby PD-L1 overexpression attenuates the effects of EGFR–TKIs in EGFR-mutant LUAD cells is depicted schematically in Fig. 8. PD-L1 overexpression could cause hyperactivation of the MAPK/ERK signaling pathway to promote cellular autophagy, which significantly promoted cell proliferation and migration and inhibited cell apoptosis, leading to gefitinib resistance. Pemetrexed synergized with gefitinib to decrease PD-L1 expression and inhibit MAPK/ERK signaling to reduce autophagy, thereby reversing the above resistance. Taken together, our results may provide a novel treatment strategy based on the expression of PD-L1 for patients with EGFR-mutant LUAD. When PD-L1 expression is negative, EGFR–TKIs monotherapy is recommended, whereas patients with PD‐L1-positive expression are preferentially considered for combination therapy with chemotherapy.

## Discussion

EGFR–TKIs resistance is a major cause for tumor recurrence and therapeutic failure in EGFR-mutant NSCLC. The molecular mechanisms of primary resistance to EGFR–TKIs are complex and still poorly understood. Recent studies have shown that PD-L1 expression is associated with EGFR mutations and the efficacy of EGFR–TKIs [45]. High PD-L1 expression may be one of the mechanisms of primary resistance to EGFR–TKIs. Many retrospective clinical studies have found that the expression of PD-L1 is significantly higher in patients with primary resistance to EGFR–TKIs, and high PD-L1 expression is correlated with shorter PFS in EGFR-mutant NSCLC [30, 46]. High PD-L1 expression has been implicated in primary resistance to EGFR–TKIs, which may induce epithelial–mesenchymal transition (EMT) through activation of the TGF-β/Smad signaling pathway [11]. In the present study, we found that positive PD-L1 expression adversely affected the prognosis of patients receiving EGFR–TKIs, which was consistent with previous studies [31, 46, 47]. We further demonstrated that PD-L1-positive expression was an independent adverse prognostic factor for PFS and could be used as a predictor of the antitumor effect of EGFR–TKIs. Previous clinical studies have found that chemotherapy combined with EGFR–TKIs is superior to TKIs alone in PFS [44]. However, other studies failed to find a significant improvement, which may be related to the different inclusion criteria [21]. Therefore, it is crucial to identify which patients are suitable for EGFR–TKIs monotherapy and which should be given combination treatment. However, there is a lack of validated prognostic biomarkers for efficiently guiding treatment selection. In the present study, subgroup analysis showed that PD-L1-positive patients rather than PD-L1-negative patients could benefit from combination therapy, which suggested that EGFR–TKIs monotherapy may be sufficient to achieve response for PD-L1-negative patients, whereas combination therapy may be the optimal treatment strategy for PD-L1-positive patients. We propose that PD-L1 can be used as a biomarker to predict poor response to EGFR–TKIs and to help select patients for a combination approach in EGFR-mutant LUAD. However, given the limited number of patients in our study, further studies with a larger population and longer follow-up are required to confirm our findings. Based on these results, we performed experiments in vitro and in vivo to explore the underlying mechanisms. We showed that overexpression of PD-L1 could facilitate the proliferation, migration and invasion of EGFR-mutant LUAD cells. In addition, PD-L1 decreased gefitinib sensitivity by promoting autophagy and inhibiting apoptosis via the MAPK/ERK signaling pathway, revealing a novel mechanism underlying PD-L1-mediated drug resistance to EGFR–TKIs.

Several studies have reported that PD-L1 promotes immune-independent tumor cell proliferation, metastasis, autophagy, drug resistance, and cancer stemness [25, 48]. Previous studies have found that PD-L1 has anti-apoptotic effects [49]. Apoptosis is a gene-regulated progression of programmed cell death, which is essential for maintaining tissue balance and cell integrity. Our study demonstrated that overexpression of PD-L1 increased autophagy and decreased apoptosis, suggesting that the increase in the proliferation of EGFR-mutant LUAD cells caused by PD-L1 overexpression was partly due to the inhibition of apoptosis.

In addition to apoptosis, autophagy, as a survival mechanism under hypoxia, starvation, and other stress conditions, sequesters cytoplasm and organelles by forming double-membrane vesicles (autophagosomes) and degrades cellular contents to maintain intracellular homeostasis [50]. Activation of autophagy may be associated with hypoxia caused by high proliferation rate, poor vascularization of tumor cells, and selective pressure of drug therapy. Autophagy may confer survival advantages to tumor cells under metabolic stress and induce resistance to therapy [51, 52]. Inhibition of autophagy may improve the sensitivity of tumor cells to radiotherapy and chemotherapy. Autophagy and apoptosis may be triggered by common upstream signals, leading to crosstalk between them: apoptosis is promoted when autophagy is inhibited [53]. Beclin1 is cleaved by caspase 3, thereby destroying its pro-autophagic activity and inducing Beclin 1-C to delocalization to mitochondria, accelerating apoptosis [54]. Although the role of PD-L1 in tumorigenesis has been extensively studied, few studies have linked PD-L1 to autophagy. In this study, we found that PD-L1 stimulated autophagy by interfering with the expression of Beclin1, p62, Atg5, Atg7 and LC3B, key regulators of autophagy, which was consistent with recent reports [55]. Furthermore, the cell proliferation and autophagy of PD-L1-overexpression cells were significantly inhibited when treated with the autophagy inhibitor 3-MA, resulting in increased apoptosis. We concluded that autophagy contributed to PD-L1-induced proliferation and regulated apoptosis in LUAD cells.

Several signaling transduction pathways have been shown to regulate the induction of autophagy, such as mTORC1, AMPK and MAPK [41, 56]. However, the mechanisms involved remain poorly understood. In our study, we found that the expression of p-ERK was significantly correlated with PD-L1 expression. ERK is a member of the MAPK family and plays a critical role in regulating cell proliferation, invasion, and metastasis in LUAD cells [57]. In addition, the MAPK/ERK signaling pathway has been implicated in the regulation of autophagy [38, 58]. The MAPK/ERK signaling pathway can promote autophagy by inducing the conversion of microtubule-associated protein 1 light chain 3 (LC3)-I to LC3-II and upregulating the autophagy-related protein Beclin1 [59]. Autophagy can remove damaged mitochondria and endoplasmic reticulum and reduce excessive reactive oxygen species (ROS), thereby inhibiting nuclear DNA damage and further inhibiting cell apoptosis [60]. When tumor cells were stimulated by adverse conditions such as radiotherapy and chemotherapy, autophagy was upregulated in cancer cells, which could increase drug resistance. Our results showed that gefitinib could promote autophagy in PD-L1-overexpression cells, but not in vector cells. We concluded that PD-L1 upregulated the levels of autophagy-related proteins and enhanced the pro-autophagic effect of gefitinib in LUAD cells, thereby increasing gefitinib resistance. In general, PD-L1 could induce autophagy and inhibit apoptosis of LUAD cells through the MAPK/ERK signaling pathway, thereby decreasing the drug sensitivity of gefitinib. In addition, our study showed that gefitinib combined with pemetrexed could synergistically downregulate the expression of PD-L1 and p-ERK, inhibit cell proliferation and autophagy, and increase apoptosis in PD-L1-overexpression cells. The combination treatment was supposed to inhibit tumor growth not only by directly blocking EGFR signaling, but also by consequently restoring antitumor immune response such as PD-L1 downregulation in EGFR-mutant cells. This implies that blockade of PD-L1 maybe a promising optional treatment for PD-L1 upregulated NSCLC patients with EGFR mutation, which requires further studies to verify its specific mechanism.

In conclusion, our study investigated the mechanism of PD-L1 in primary resistance to EGFR–TKIs in EGFR-mutant LUAD cells and revealed the important role of PD-L1. Results showed that PD-L1 promoted proliferation and autophagy and inhibited the apoptosis of LUAD cells, which contributed to tumor progression and the development of gefitinib resistance. We also found that the combination of gefitinib and pemetrexed could synergistically enhance the antitumor efficacy of PD-L1-overexpression LUAD cells, providing new ideas and methods to overcome EGFR–TKIs resistance. Clarifying the prognostic value of PD-L1 expression in patients treated with EGFR–TKIs may provide guidance for selecting highly appropriate therapeutic regimens. In conclusion, the present study provides novel insights into EGFR–TKIs primary resistance and an important rationale for precision medicine treatments for patients with EGFR-mutant LUAD. It can be used as a reference for research in other cancers.

### Supplementary information

Supplementary Material

Original western blots

## Data Availability

All data generated or analyzed during this study are included in this published article and its supplementary information files.
