# Supplementary material for: PD-L1 induces autophagy and primary resistance to EGFR–TKIs in EGFR-mutant lung adenocarcinoma via the MAPK signaling pathway
Source: Cell Death Dis. 2024 Aug 1;15(8):555. doi: 10.1038/s41419-024-06945-7 (PMC11294607; doi:10.1038/s41419-024-06945-7)
Supplement: Supplementary file 1 — Supplementary Material [file 41419_2024_6945_MOESM1_ESM.pdf]

Supplementary information

Supplementary Figure 1. Gene ontology (GO) enrichment analysis of differentially expressed genes (DEGs).

Supplementary Figure 2. The effects of PD-L1 overexpression on p-p38, p-JNK, p-AKT and p-NF-κB proteins levels were verified by Western Blotting in PC9 and HCC827 cells.

Supplementary Figure 3. A. The effects of PD-L1 overexpression on EGFR downstream signaling pathway activation were verified by Western Blotting in EGFR-wild type H1299 cells. B. The protein levels of p-ERK, ERK, apoptosis and autophagy-related proteins were assessed by Western Blotting. H1299 PD-L1 cells were pretreated with MER/ERK pathway inhibitor PD98059. C. H1299 cells as in (B) were immunostained with antibodies against LC3B (scale bar, 20 μm). D. Apoptotic rate was measured using Annexin V/7-AAD staining in cells as in (B). E-G. The viability of cells as in (B) was assessed by colony formation assay (E), EDU assay (F) and CCK-8 assay (G) (scale bar, 150 μm). H, I. Cell migration and invasion of H1299 PD-L1 and vector cells were measured by Transwell assay. The different cells were pretreated with gefitinib, pemetrexed or combined treatment for 72 h (scale bar, 150 μm; magnification, 20 ×). J. Wound-healing assay comparing the cell migration differences among H1299 PD-L1 and vector cells under gefitinib, pemetrexed or combined treatment for 24 h (scale bar, 750 μm). The analyses were repeated three times. Data were presented as mean ± SD. *$p < 0.05$, **$p < 0.01$, ***$p < 0.001$, ****$p < 0.0001$

Supplementary Figure 4. A. PC9 PD-L1 and vector cells were treated with different

combinations of concentrations of gefitinib and pemetrexed for 72 h, and the CI values of gefitinib-pemetrexed combination therapy were calculated. B. HCC827 PD-L1 and vector cells were treated with different combinations of concentrations of gefitinib and pemetrexed for 72 h, and the CI values of gefitinib-pemetrexed combination therapy were calculated. CI, Combination index.

Supplementary Table 1. Univariate and multivariate analysis for ORR in EGFR–TKIs monotherapy cohort.

Characteristics	Objective responses (complete and partial) (n)	Patients (n)	ORR (%)	Univariate analysis		Multivariate analysis	
				Odds ratio (95% CI)	*p*	Odds ratio (95 % CI)	*p*
Sex							
male	13	32	40.6	0.489	0.147	0.324	0.125
female	21	36	58.3	(0.186-1.286)		(0.077-1.367)	
Age							
< 60	16	34	47.1	0.790	0.628	1.141	0.813
≥ 60	18	34	52.9	(0.305-2.048)		(0.383-3.400)	
Smoking history							
no	23	45	51.1	1.140	0.798	0.567	0.469
yes	11	23	47.8	(0.417-3.117)		(0.122-2.636)	
TNM stage							
III	8	11	72.7	3.179	0.112	3.626	0.112
IV	26	57	45.6	(0.764-13.228)		(0.741-17.737)	
PD-L1status							
negative	16	22	72.7	4.148	0.012*	4.170	0.017*
positive	18	46	39.1	(1.368-12.580)		(1.294-13.437)	

Abbreviations: ORR, Objective response rate; CI, confidence interval. *$p < 0.05$

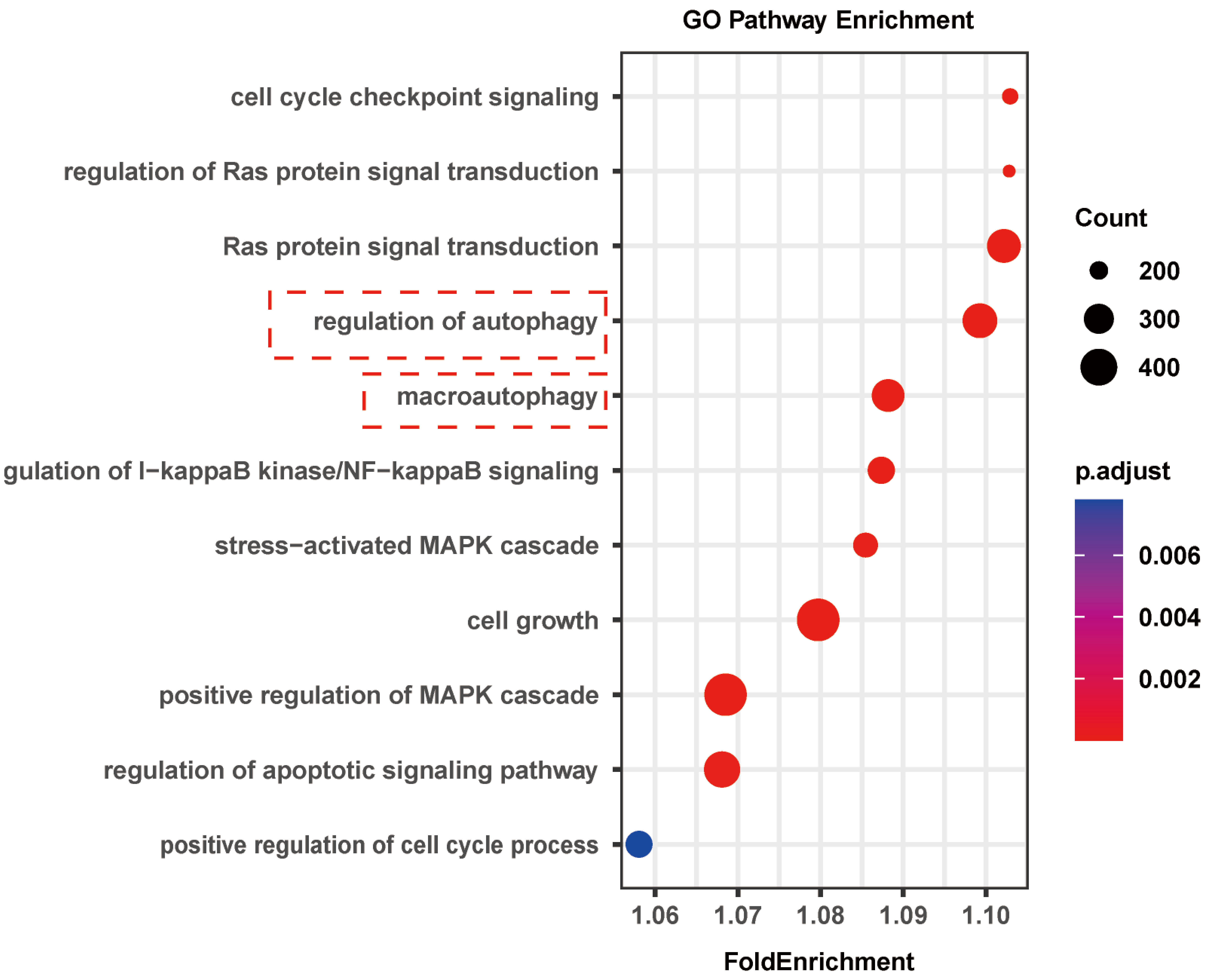
GO Pathway Enrichment
cell cycle checkpoint signaling
regulation of Ras protein signal transduction
Ras protein signal transduction
regulation of autophagy
macroautophagy
gulation of I−kappaB kinase/NF−kappaB signaling
stress−activated MAPK cascade
cell growth
positive regulation of MAPK cascade
regulation of apoptotic signaling pathway
positive regulation of cell cycle process
1.06
1.07
1.08
1.09
1.10
FoldEnrichment
Count
200
300
400
p.adjust
0.006
0.004
0.002

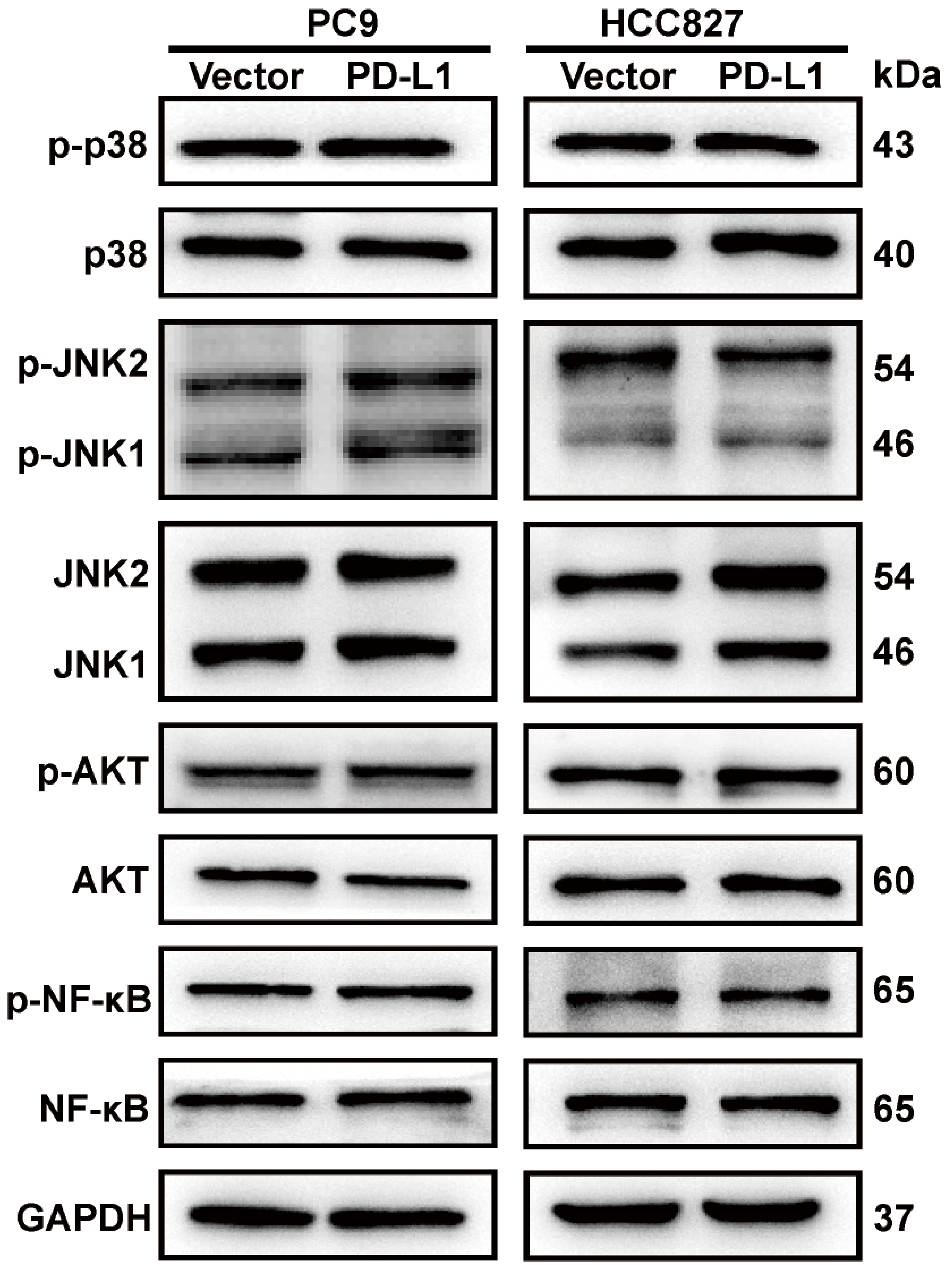
PC9
HCC827
Vector
PD-L1
Vector
PD-L1
kDa
p-p38
43
p38
40
p-JNK2
54
p-JNK1
46
JNK2
54
JNK1
46
p-AKT
60
AKT
60
p-NF-κB
65
NF-κB
65
GAPDH
37

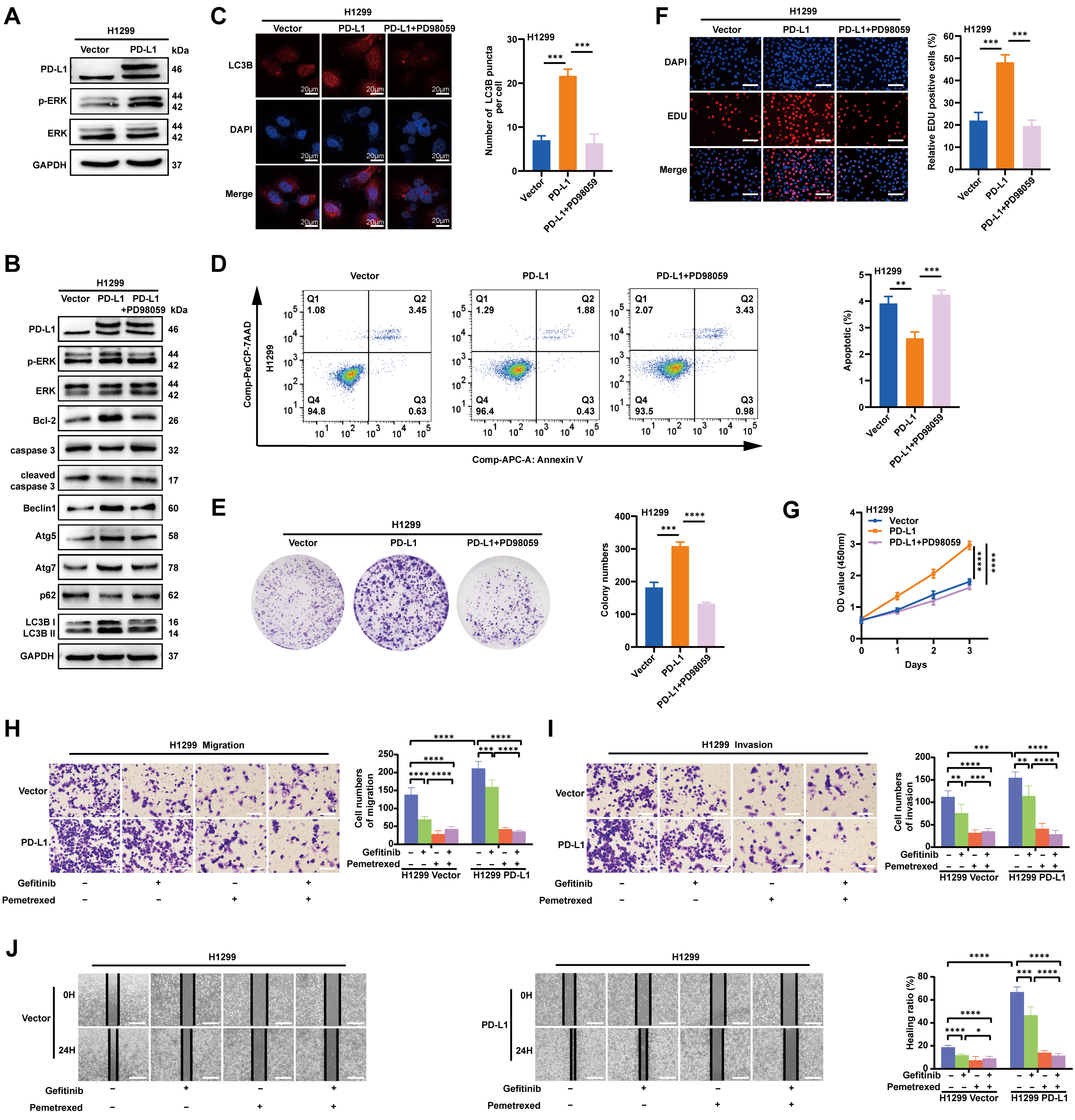
A
H1299
Vector
PD-L1
kDa
PD-L1
p-ERK
ERK
GAPDH
46
44
42
37
B
PD-L1+PD98059
Bcl-2
caspase 3
cleaved caspase 3
Beclin1
Atg5
Atg7
p62
LC3B I
LC3B II
C
LC3B
DAPI
Merge
20μm
Number of LC3B puncta per cell
D
Comp-PerCP-7AAD
Comp-APC-A: Annexin V
Apoptotic (%)
E
Colony numbers
F
EDU
Relative EDU positive cells (%)
G
OD value (450nm)
Days
H
H1299 Migration
Gefitinib
Pemetrexed
Cell numbers of migration
H1299 Vector
H1299 PD-L1
I
H1299 Invasion
Cell numbers of invasion
J
0H
24H
Healing ratio (%)

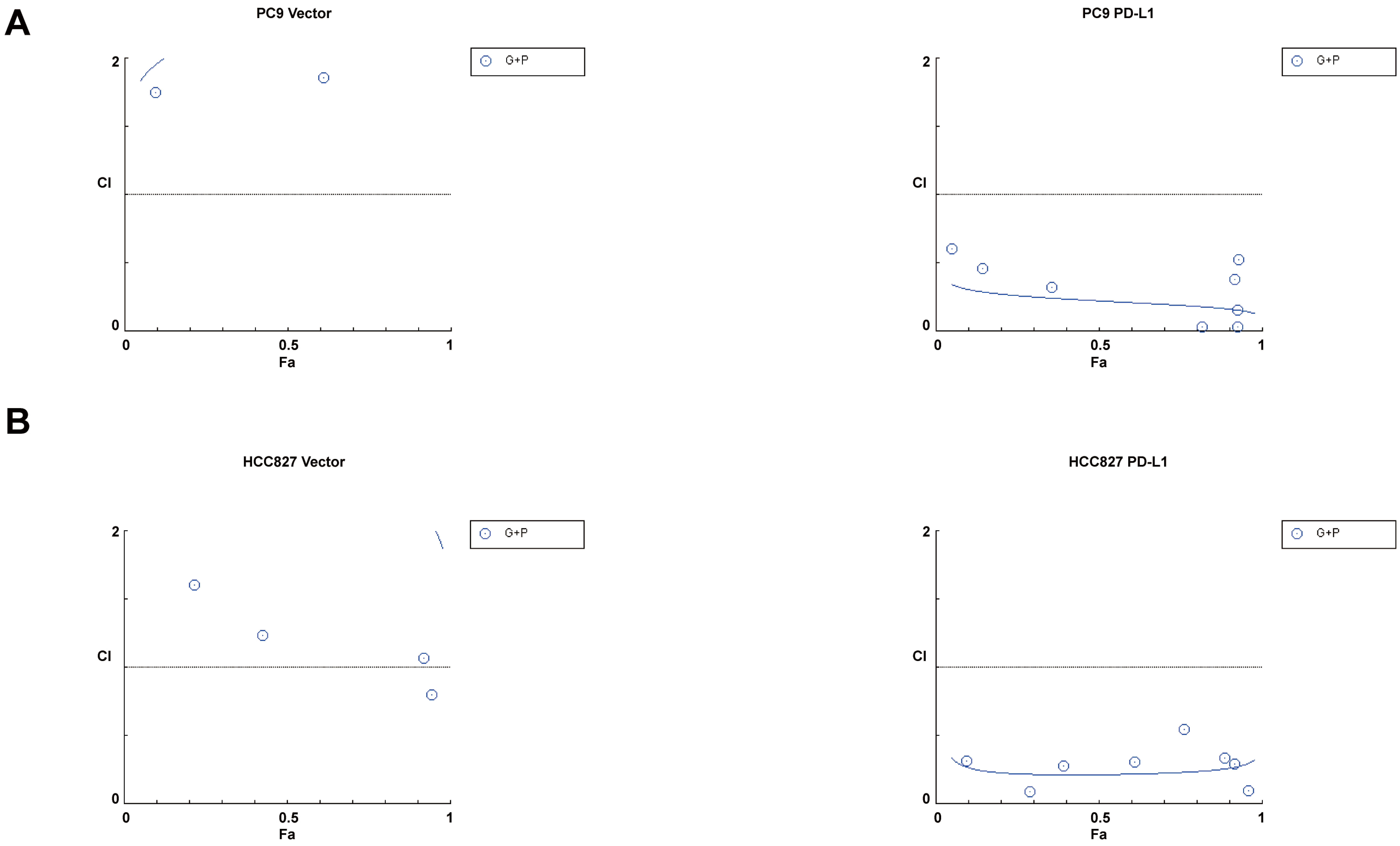
A
PC9 Vector
PC9 PD-L1
B
HCC827 Vector
HCC827 PD-L1
G+P
CI
Fa
2
0
0
0.5
1
