# Supplementary material for: PD-L1 induces autophagy and primary resistance to EGFR–TKIs in EGFR-mutant lung adenocarcinoma via the MAPK signaling pathway
Source: Cell Death Dis. 2024 Aug 1;15(8):555. doi: 10.1038/s41419-024-06945-7 (PMC11294607; doi:10.1038/s41419-024-06945-7)

Original western blots

Fig. 2A

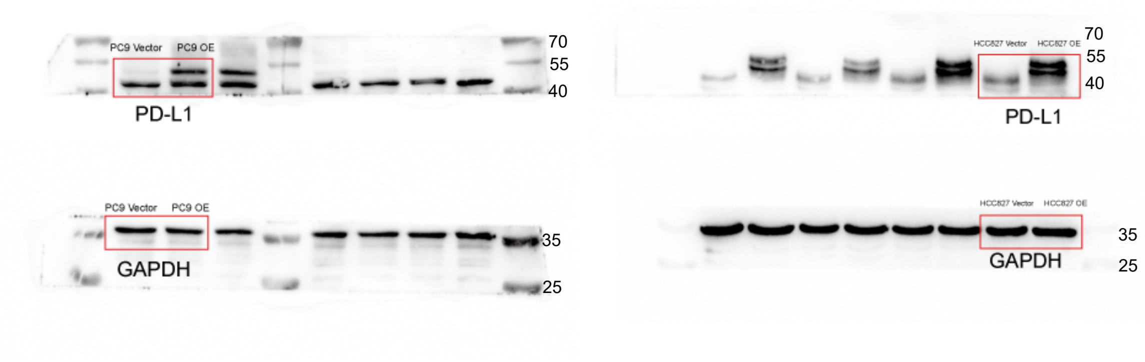

Fig. 2G

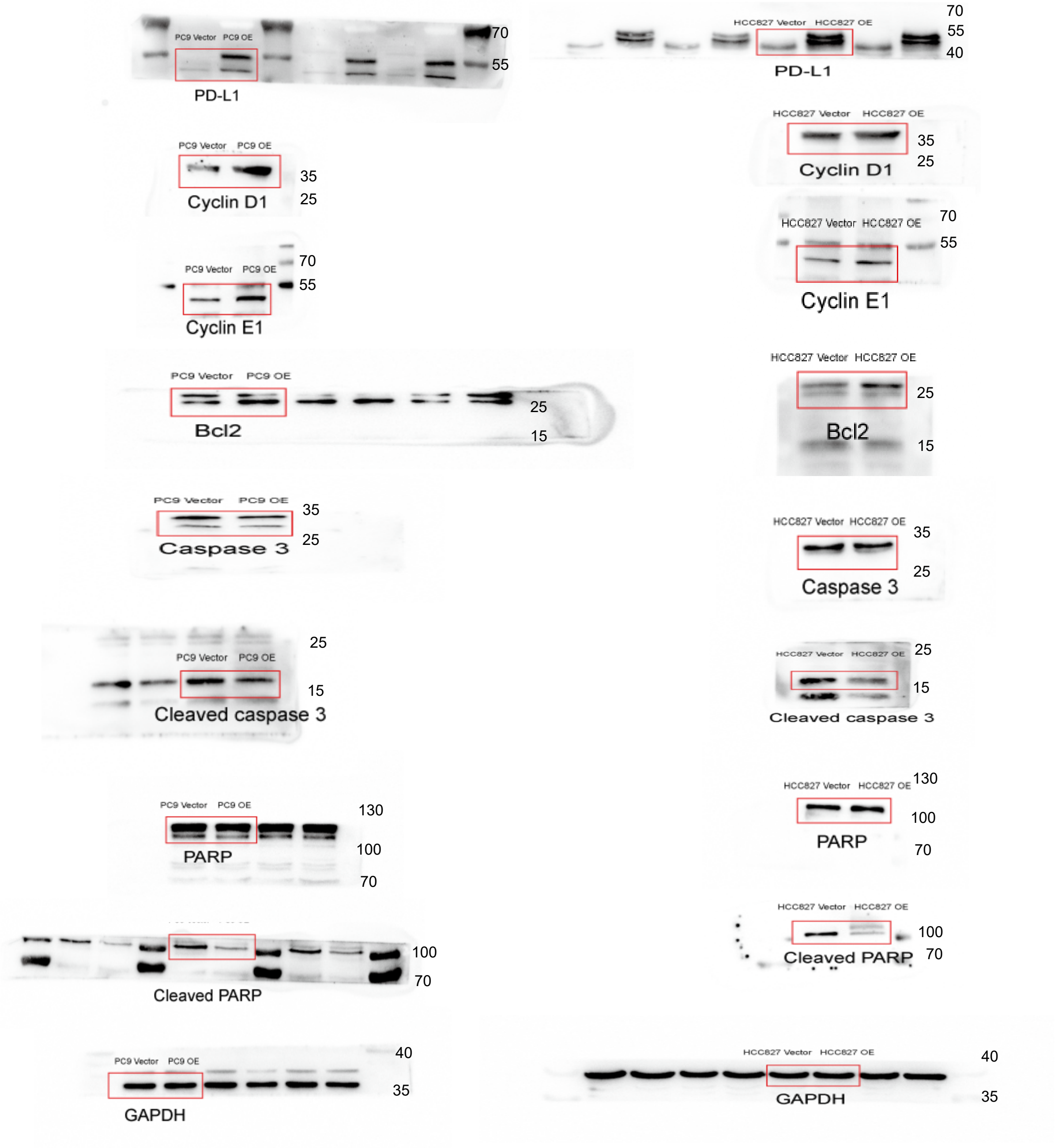

Fig. 3D

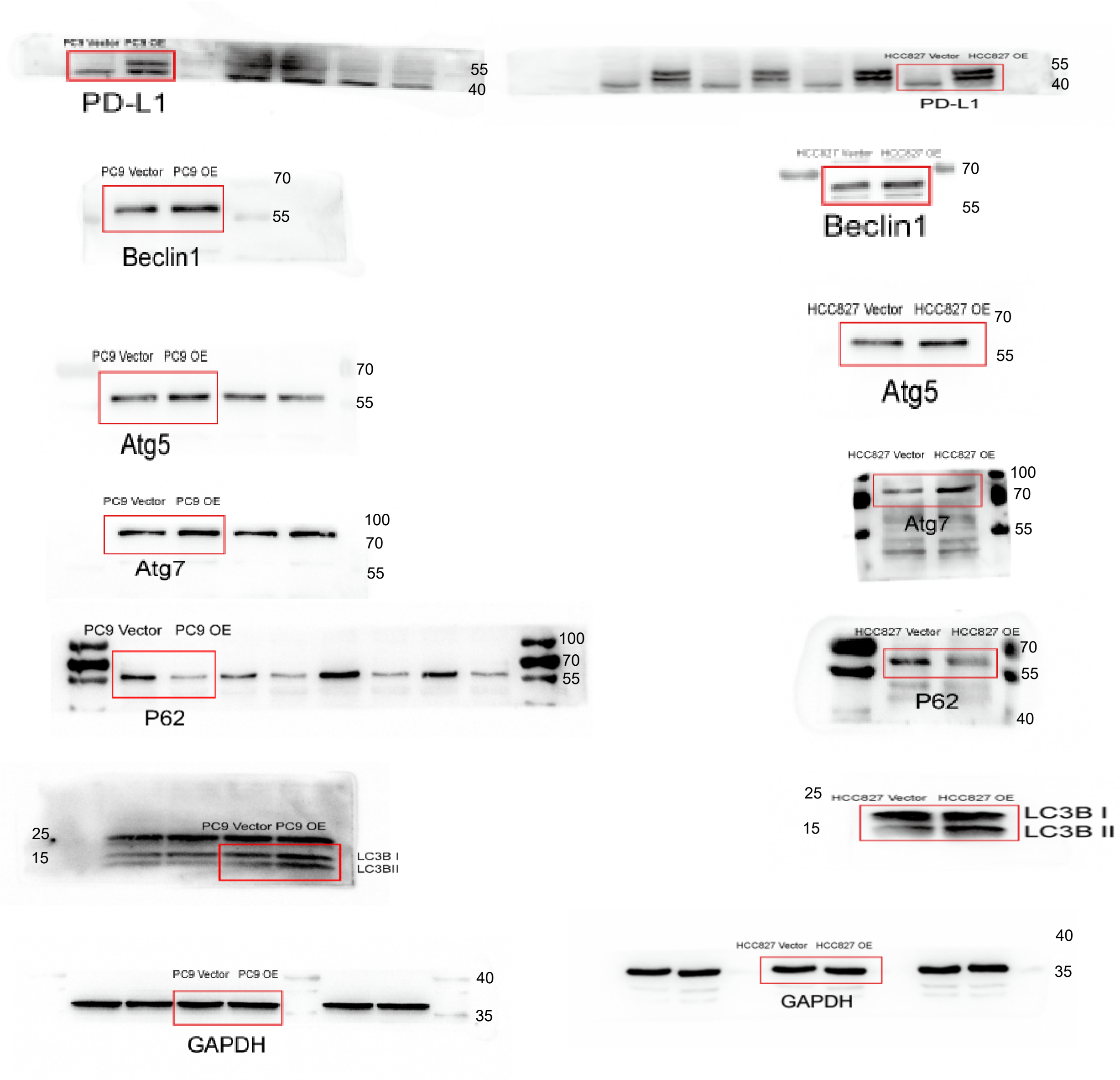
PC9 Vector PC9 OE
55
40
PD-L1
HCC827 Vector HCC827 OE
55
40
PD-L1
PC9 Vector PC9 OE
70
55
Beclin1
70
55
Beclin1
HCC827 Vector HCC827 OE
70
55
Atg5
PC9 Vector PC9 OE
70
55
Atg5
HCC827 Vector HCC827 OE
100
70
Atg7
55
PC9 Vector PC9 OE
100
70
Atg7
55
PC9 Vector PC9 OE
100
70
55
P62
HCC827 Vector HCC827 OE
70
55
P62
40
PC9 Vector PC9 OE
25
15
LC3B I
LC3BII
25
HCC827 Vector HCC827 OE
15
LC3B I
LC3B II
PC9 Vector PC9 OE
40
35
GAPDH
HCC827 Vector HCC827 OE
40
35
GAPDH

Fig. 3I

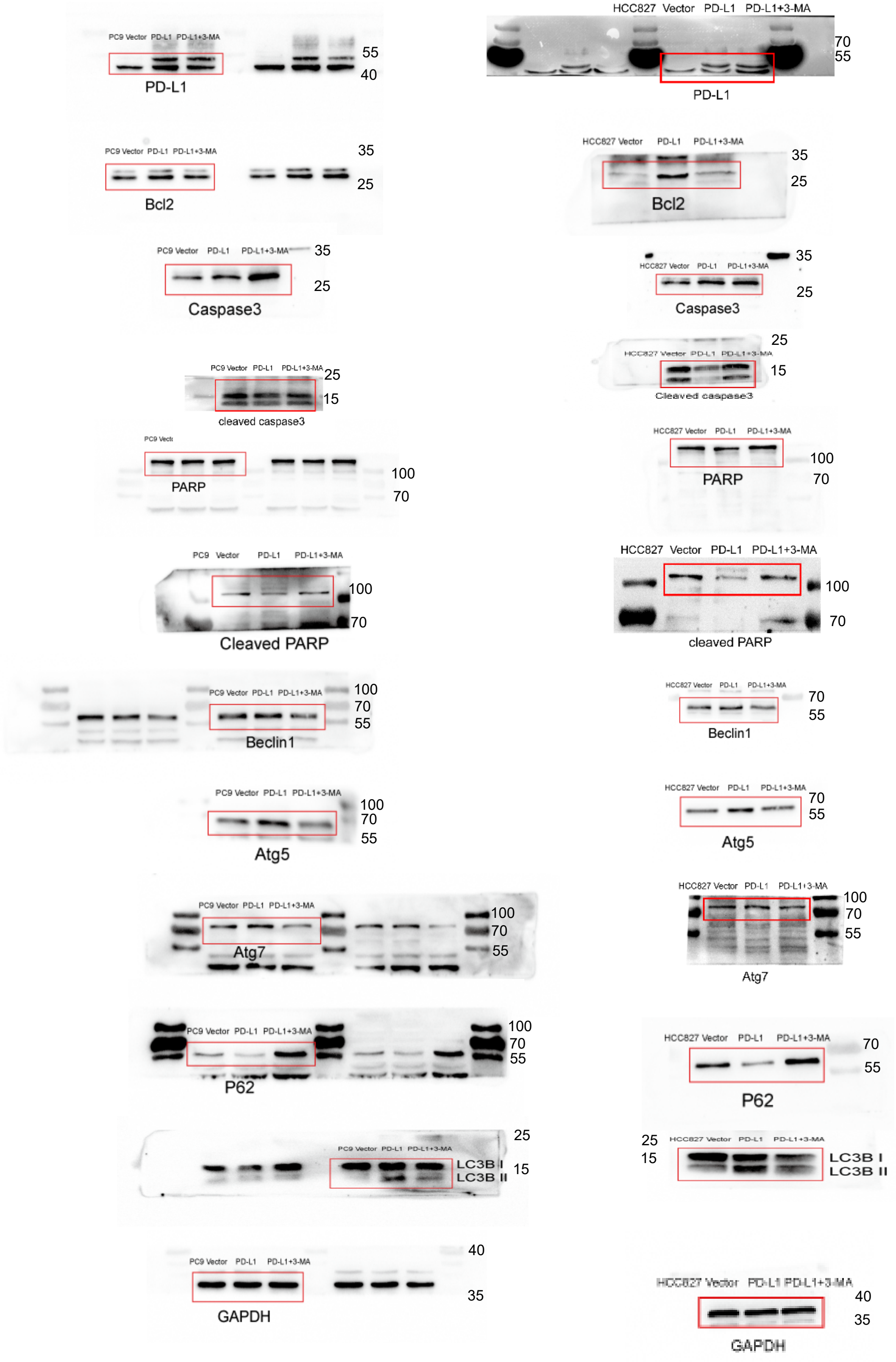

PC9 Vector PD-L1 PD-L1+3-MA
PD-L1
55
40
Bcl2
35
25
Caspase3
cleaved caspase3
15
PARP
100
70
Cleaved PARP
Beclin1
Atg5
Atg7
P62
LC3B I
LC3B II
GAPDH
HCC827 Vector PD-L1 PD-L1+3-MA
Cleaved caspase3
cleaved PARP

Fig. 4A

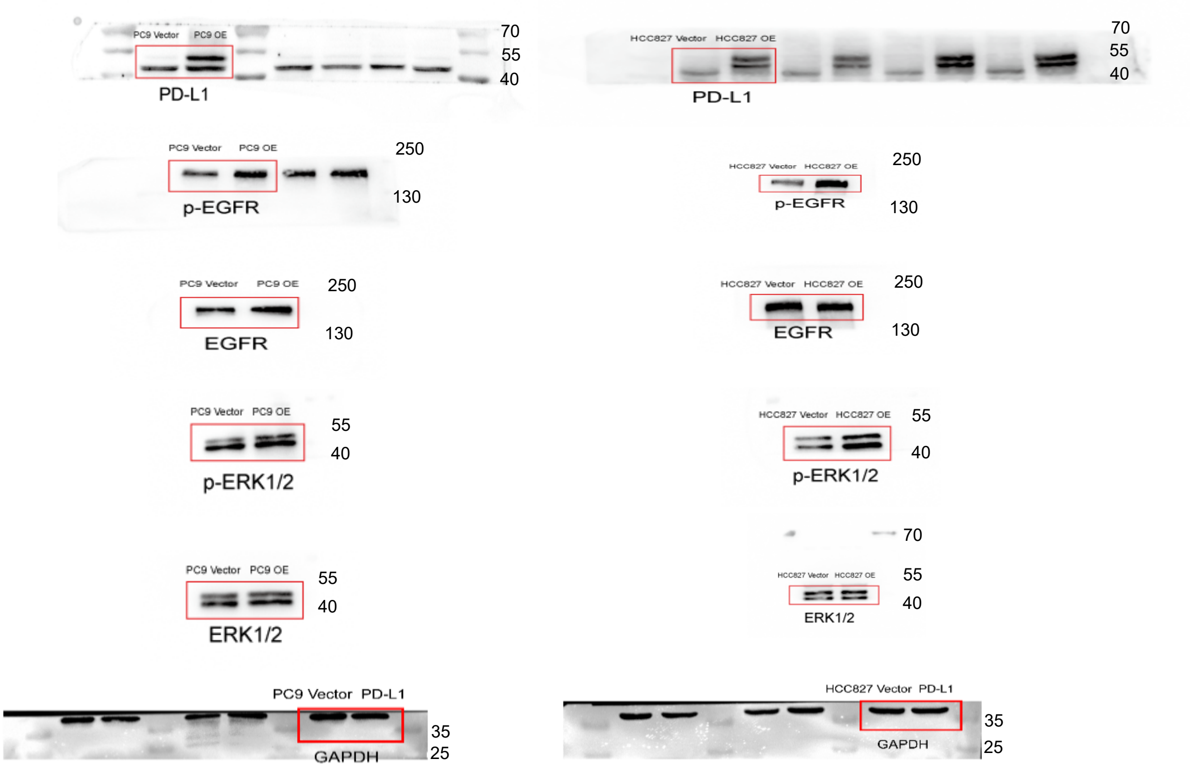
PC9 Vector
PC9 OE
70
55
40
PD-L1
HCC827 Vector
HCC827 OE
70
55
40
PD-L1
PC9 Vector
PC9 OE
250
130
p-EGFR
HCC827 Vector
HCC827 OE
250
p-EGFR
130
PC9 Vector
PC9 OE
250
130
EGFR
HCC827 Vector
HCC827 OE
250
EGFR
130
PC9 Vector
PC9 OE
55
40
p-ERK1/2
HCC827 Vector
HCC827 OE
55
40
p-ERK1/2
70
PC9 Vector
PC9 OE
55
40
ERK1/2
55
40
ERK1/2
PC9 Vector
PD-L1
35
GAPDH
25
HCC827 Vector
PD-L1
35
GAPDH
25

Fig. 4B

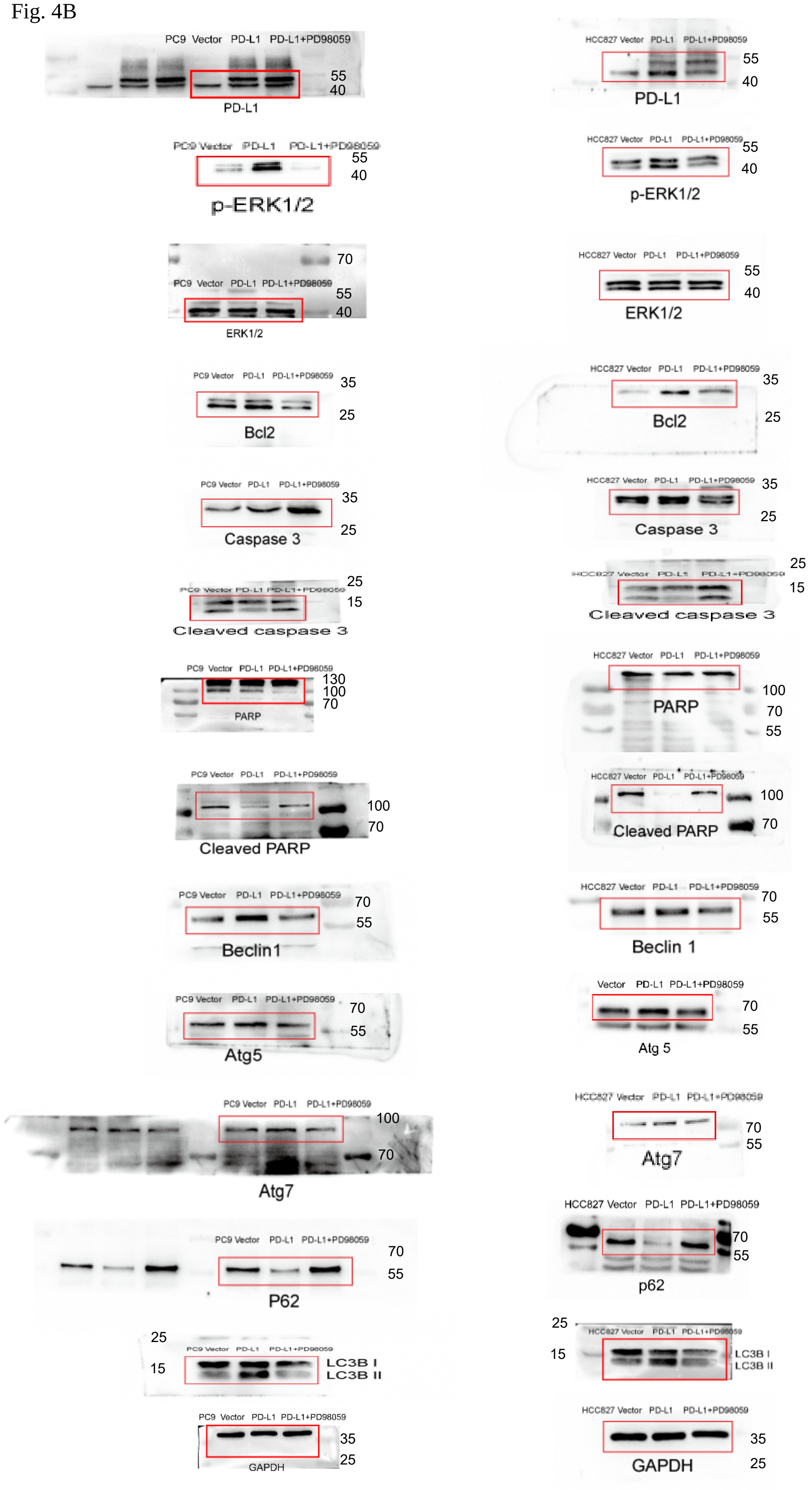

PC9 Vector PD-L1 PD-L1+PD98059
55
40
PD-L1
PC9 Vector PD-L1 PD-L1+PD98059
55
40
p-ERK1/2
70
PC9 Vector PD-L1 PD-L1+PD98059
55
40
ERK1/2
PC9 Vector PD-L1 PD-L1+PD98059
35
25
Bcl2
PC9 Vector PD-L1 PD-L1+PD98059
35
25
Caspase 3
25
PC9 Vector PD-L1 PD-L1+PD98059
15
Cleaved caspase 3
PC9 Vector PD-L1 PD-L1+PD98059
130
100
70
PARP
PC9 Vector PD-L1 PD-L1+PD98059
100
70
Cleaved PARP
PC9 Vector PD-L1 PD-L1+PD98059
70
55
Beclin1
PC9 Vector PD-L1 PD-L1+PD98059
70
55
Atg5
PC9 Vector PD-L1 PD-L1+PD98059
100
70
Atg7
PC9 Vector PD-L1 PD-L1+PD98059
70
55
P62
25
PC9 Vector PD-L1 PD-L1+PD98059
15
LC3B I
LC3B II
PC9 Vector PD-L1 PD-L1+PD98059
35
25
GAPDH
HCC827 Vector PD-L1 PD-L1+PD98059
55
40
PD-L1
HCC827 Vector PD-L1 PD-L1+PD98059
55
40
p-ERK1/2
HCC827 Vector PD-L1 PD-L1+PD98059
55
40
ERK1/2
HCC827 Vector PD-L1 PD-L1+PD98059
35
Bcl2
25
HCC827 Vector PD-L1 PD-L1+PD98059
35
25
Caspase 3
25
HCC827 Vector PD-L1 PD-L1+PD98059
15
Cleaved caspase 3
HCC827 Vector PD-L1 PD-L1+PD98059
100
PARP
70
55
HCC827 Vector PD-L1 PD-L1+PD98059
100
70
Cleaved PARP
HCC827 Vector PD-L1 PD-L1+PD98059
70
55
Beclin 1
Vector PD-L1 PD-L1+PD98059
70
55
Atg 5
HCC827 Vector PD-L1 PD-L1+PD98059
70
55
Atg7
HCC827 Vector PD-L1 PD-L1+PD98059
70
55
p62
25
HCC827 Vector PD-L1 PD-L1+PD98059
15
LC3B I
LC3B II
HCC827 Vector PD-L1 PD-L1+PD98059
35
25
GAPDH

Fig. 6I

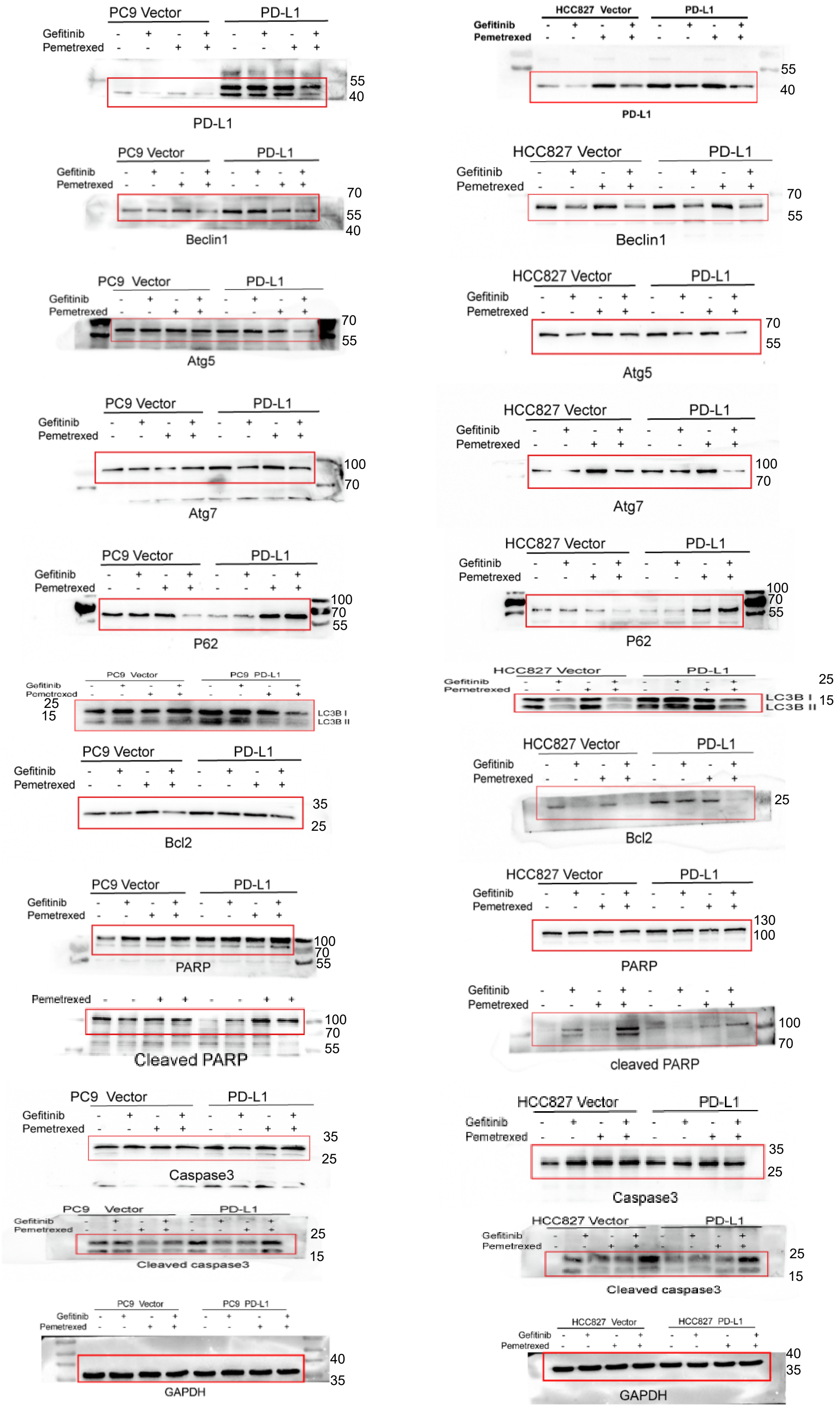

PC9 Vector
PD-L1
Gefitinib
Pemetrexed
55
40
PD-L1
HCC827 Vector
PD-L1
Gefitinib
Pemetrexed
55
40
PD-L1
PC9 Vector
PD-L1
70
55
40
Beclin1
HCC827 Vector
PD-L1
70
55
Beclin1
PC9 Vector
PD-L1
70
55
Atg5
HCC827 Vector
PD-L1
70
55
Atg5
PC9 Vector
PD-L1
100
70
Atg7
HCC827 Vector
PD-L1
100
70
Atg7
PC9 Vector
PD-L1
100
70
55
P62
HCC827 Vector
PD-L1
100
70
55
P62
PC9 Vector
PC9 PD-L1
25
15
LC3B I
LC3B II
HCC827 Vector
PD-L1
25
15
LC3B I
LC3B II
PC9 Vector
PD-L1
35
25
Bcl2
HCC827 Vector
PD-L1
25
Bcl2
PC9 Vector
PD-L1
100
70
55
PARP
HCC827 Vector
PD-L1
130
100
PARP
Pemetrexed
100
70
55
Cleaved PARP
100
70
cleaved PARP
PC9 Vector
PD-L1
35
25
Caspase3
HCC827 Vector
PD-L1
35
25
Caspase3
PC9 Vector
PD-L1
25
15
Cleaved caspase3
HCC827 Vector
PD-L1
25
15
Cleaved caspase3
PC9 Vector
PC9 PD-L1
40
35
GAPDH
HCC827 Vector
HCC827 PD-L1
40
35
GAPDH

Fig. 6J

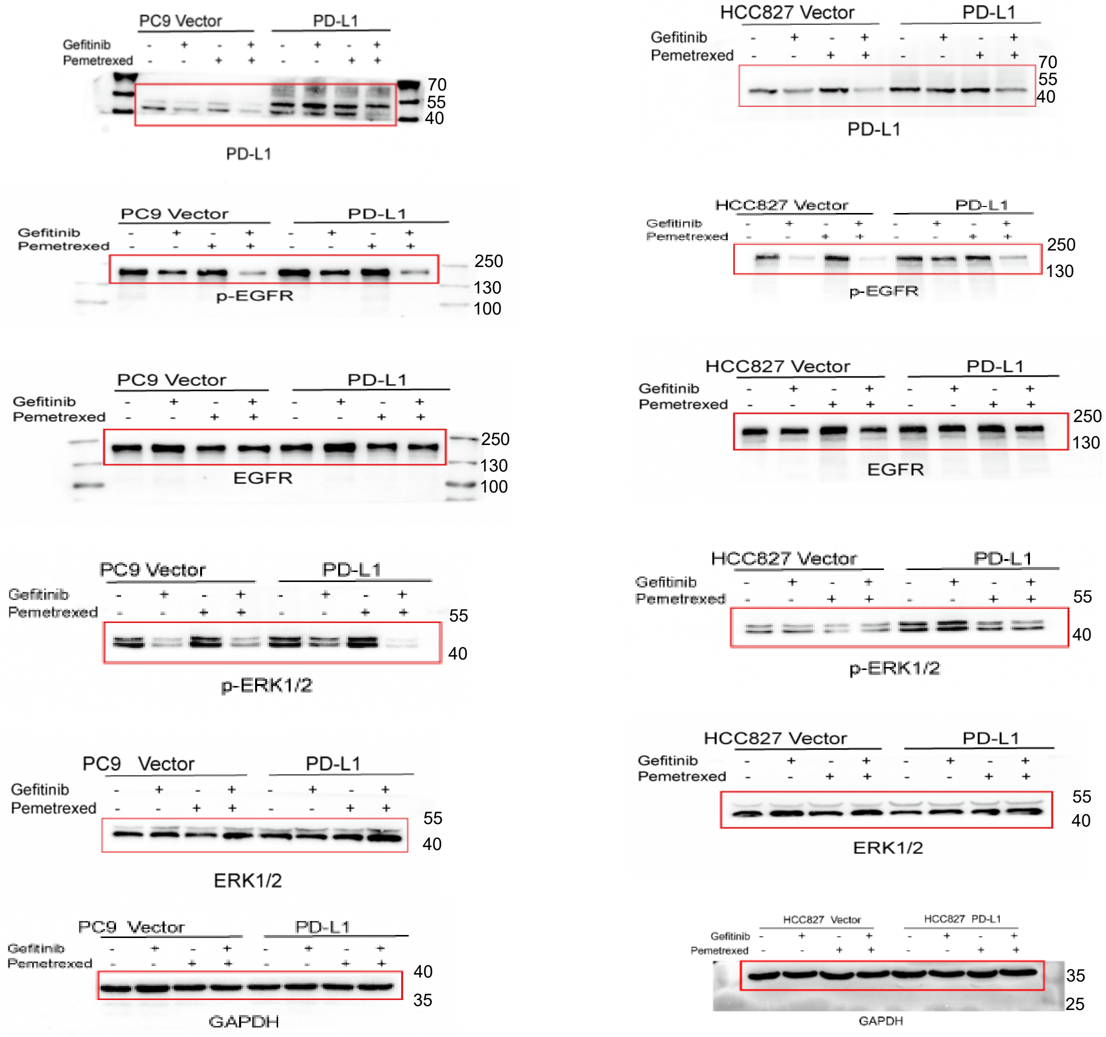
PC9 Vector
PD-L1
Gefitinib
Pemetrexed
70
55
40
PD-L1
p-EGFR
250
130
100
EGFR
p-ERK1/2
ERK1/2
GAPDH
35
HCC827 Vector
HCC827 PD-L1
25

Supplementary Figure 2

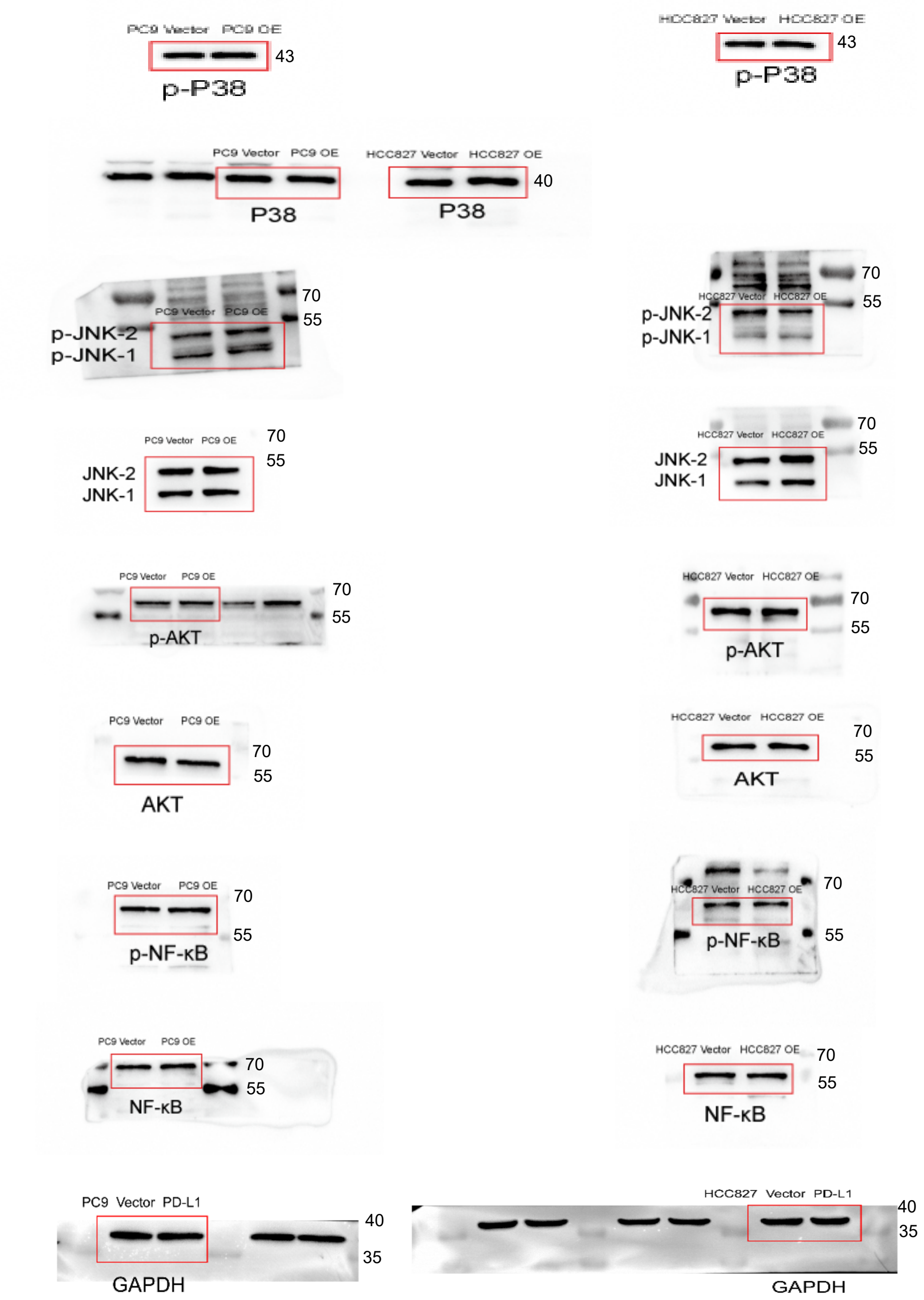

Supplementary Figure 3A

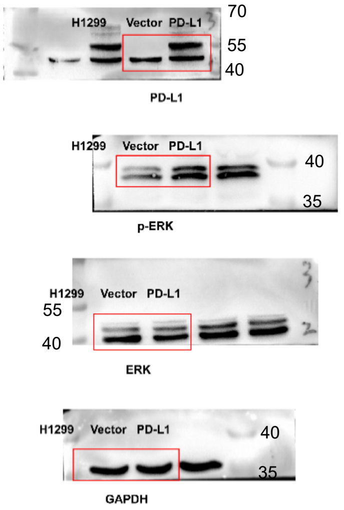

Supplementary Figure 3B

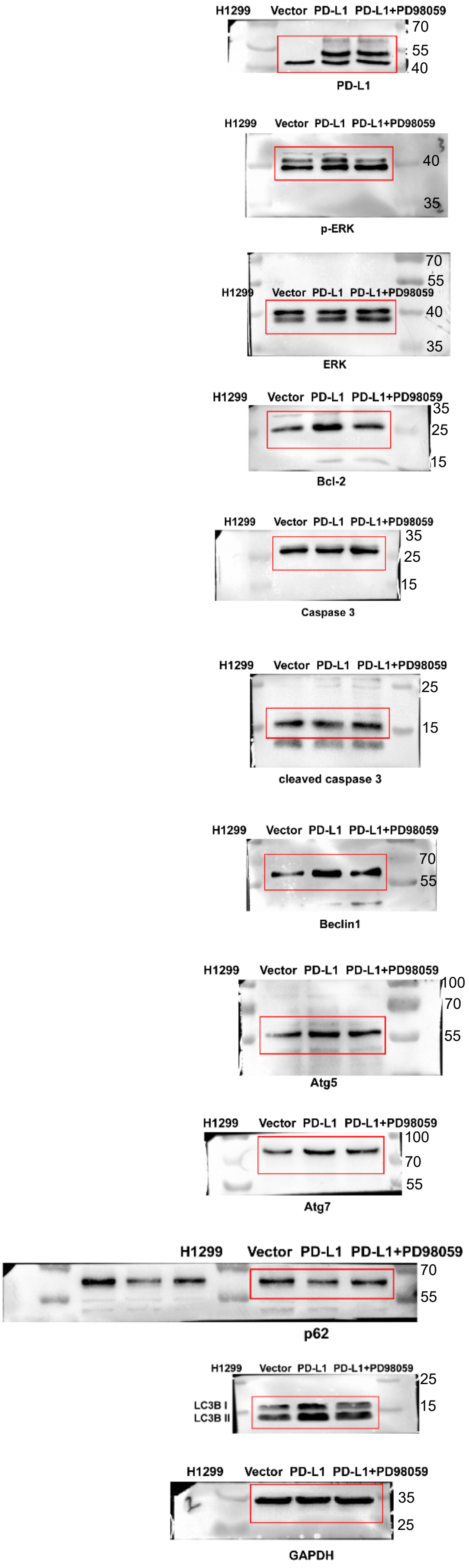

Supplement: Supplementary file 2 — Original western blots [file 41419_2024_6945_MOESM2_ESM.pdf]
